# Targeting the host factor HGS–viral membrane protein interaction in coronavirus infection

**DOI:** 10.1172/JCI200225

**Published:** 2025-12-16

**Authors:** Xubing Long, Rongrong Chen, Rong Bai, Buyun Tian, Yu Cao, Kangying Chen, Fuyu Li, Yiliang Wang, Yongjie Tang, Qi Yang, Liping Ma, Fan Wang, Maoge Zhou, Xianjie Qiu, Yongzhi Lu, Jie Zheng, Peng Zhou, Xinwen Chen, Qian Liu, Xuepeng Wei, Yongxia Shi, Yanhong Xue, Jincun Zhao, Wei Ji, Liqiao Hu, Jinsai Shang, Tao Xu, Zonghong Li

**Affiliations:** 1 The First Affiliated Hospital of Guangzhou Medical University, Guangzhou Medical University, Guangzhou, Guangdong, China.; 2 Guangzhou National Laboratory, Guangzhou, China.; 3 Key Laboratory of Biomacromolecules (CAS), National Laboratory of Biomacromolecules, Institute of Biophysics, Chinese Academy of Sciences, Beijing, China.; 4 College of Life Sciences, University of Chinese Academy of Sciences, Beijing, China.; 5 Wuhan Institute of Virology, Chinese Academy of Sciences, Wuhan, China.; 6 State Key Laboratory of Respiratory Disease, Health Quarantine Institute of IQTC, Guangzhou Customs District, Guangzhou, China.; 7 Shanghai Institute of Virology, Shanghai Jiao Tong University School of Medicine, Shanghai, China.

**Keywords:** Cell biology, Microbiology, Virology, COVID-19, Drug screens, Mouse models

## Abstract

While current antivirals primarily target viral proteins, host-directed strategies remain underexplored. Here, we performed a genome-wide CRISPR inhibition (CRISPRi) screening to identify the host protein, hepatocyte growth factor-regulated tyrosine kinase substrate (HGS), facilitating the pan-coronavirus infection both in vitro and in vivo. Mechanistically, HGS interacts with the viral membrane (M) protein, facilitating its trafficking to the ER-Golgi intermediate compartment for virion assembly. Conversely, HGS deficiency caused M retention in the ER, blocking assembly. Leveraging this interaction, we designed M-derived peptides and screened over 5,000 FDA-approved or commonly used drugs, identifying riboflavin tetrabutyrate (RTB). Both the peptides and RTB bind HGS and disrupt its interaction with the M protein, leading to M retention in the ER and subsequent blockade of virion assembly. These agents demonstrated broad anti-pan-coronavirus activity in vitro and in vivo. Collectively, our findings establish HGS as a druggable host target and identify RTB as a promising broad-spectrum antiviral candidate.

## Abstract 2

Genome-wide CRISPRi screening identifies HGS as a druggable host factor that facilitates pan-coronavirus infection, highlighting RTB as a promising broad-spectrum antiviral candidate.

## Introduction

Host-targeting agents (HTAs) have the potential to combat a broad spectrum of viruses, including newly emerging strains, by targeting host proteins important for viral replication. This approach presents a higher genetic barrier to resistance compared with virus-targeting agents (VTAs), which directly target viral proteins, as host factors are not under the virus’s genetic control. HTAs can also simultaneously target multiple viruses, making them effective against coinfections. Notable examples of HTAs include maraviroc, an FDA-approved drug that targets the CCR5 receptor to treat HIV-1 (1); camostat, an inhibitor of TMPRSS2 and a clinically approved drug for chronic pancreatitis, which can block the entry of SARS-CoV-2 (2); and teicoplanin, a clinical antibiotic, which also inhibits coronavirus entry by blocking cathepsin L (3). However, challenges arise, such as potential cytotoxicity and side effects associated with targeting host factors, as well as the possibility of viruses developing resistance by adapting their interactions with cellular factors. Additionally, the complexity of host-virus interactions necessitates advanced tools for screening and prediction.

Genome-wide CRISPR/Cas9 screening is a powerful tool for identifying critical host factors that regulate virus infection. Most studies have utilized cytopathic effect–based, genome-wide CRISPR/Cas9 screening to identify key host factors involved in virus cell cycles (4–8). In the context of infection, insights are still mostly confined to early steps in the viral cycle, predominantly viral entry and replication. However, the identification of host factors involved in regulating virus assembly and egress through cytopathic effect–based screens has been limited. Targeting viral assembly and release provides another major antiviral strategy, which can synergize with strategies that suppress viral genome replication, leading to more effective overall antiviral activity. Here, using coronavirus as a model system, we established a genome-wide CRISPR inhibition (CRISPRi) screening based on cell surface lysosome-associated membrane glycoprotein 1 (LAMP1) and identified a series of key host factors involved in virus assembly and egress. Subsequent research revealed hepatocyte growth factor-regulated tyrosine kinase substrate (HGS), a subunit of endosomal sorting complex required for transport (ESCRT), plays a vital role in pan-coronavirus infection in vitro and in vivo. HGS interacts with viral structural membrane (M) protein, facilitating its trafficking to the ERGIC for virion assembly. Importantly, the discovery of high-affinity peptides and a commonly used drug, riboflavin tetrabutyrate (RTB), targeting HGS have shown anti-pan-coronavirus activity in vitro, in air-liquid interface–cultured (ALI-cultured) human bronchial epithelial cells (HBEs), and in vivo, indicating the potential therapeutic significance of targeting HGS in combating coronavirus infections.

## Results

### Genome-wide CRISPRi screens identify HGS as a host target for pan-coronavirus therapy.

Numerous studies have suggested that coronavirus egress occurs through the lysosome, resulting in the upregulation of the lysosomal marker LAMP1 on the cell surface membrane (9–12). This observation led us to hypothesize that surface LAMP1 could serve as a reliable indicator of coronavirus egress. To validate this hypothesis, we utilized specific anti-LAMP1 antibodies conjugated with APC and FITC to distinguish between cell surface and total LAMP1, where total LAMP1 served as a control. Following infection with mouse hepatitis virus (MHV), the ratio of cell surface LAMP1 to total LAMP1 was significantly elevated (Supplemental Figure 1A; supplemental material available online with this article; https://doi.org/10.1172/JCI200225DS1), consistent with previous findings (9, 10). Knockdown of *Arl8b*, a known critical host factor involved in the regulation of virion egress (10), led to a reduction in the ratio of cell surface LAMP1 to total LAMP1 (Supplemental Figure 1, A and B). This outcome indicates that the ratio of cell surface LAMP1 to total LAMP1 represents a suitable indicator of coronavirus egress.

**Figure 1:**
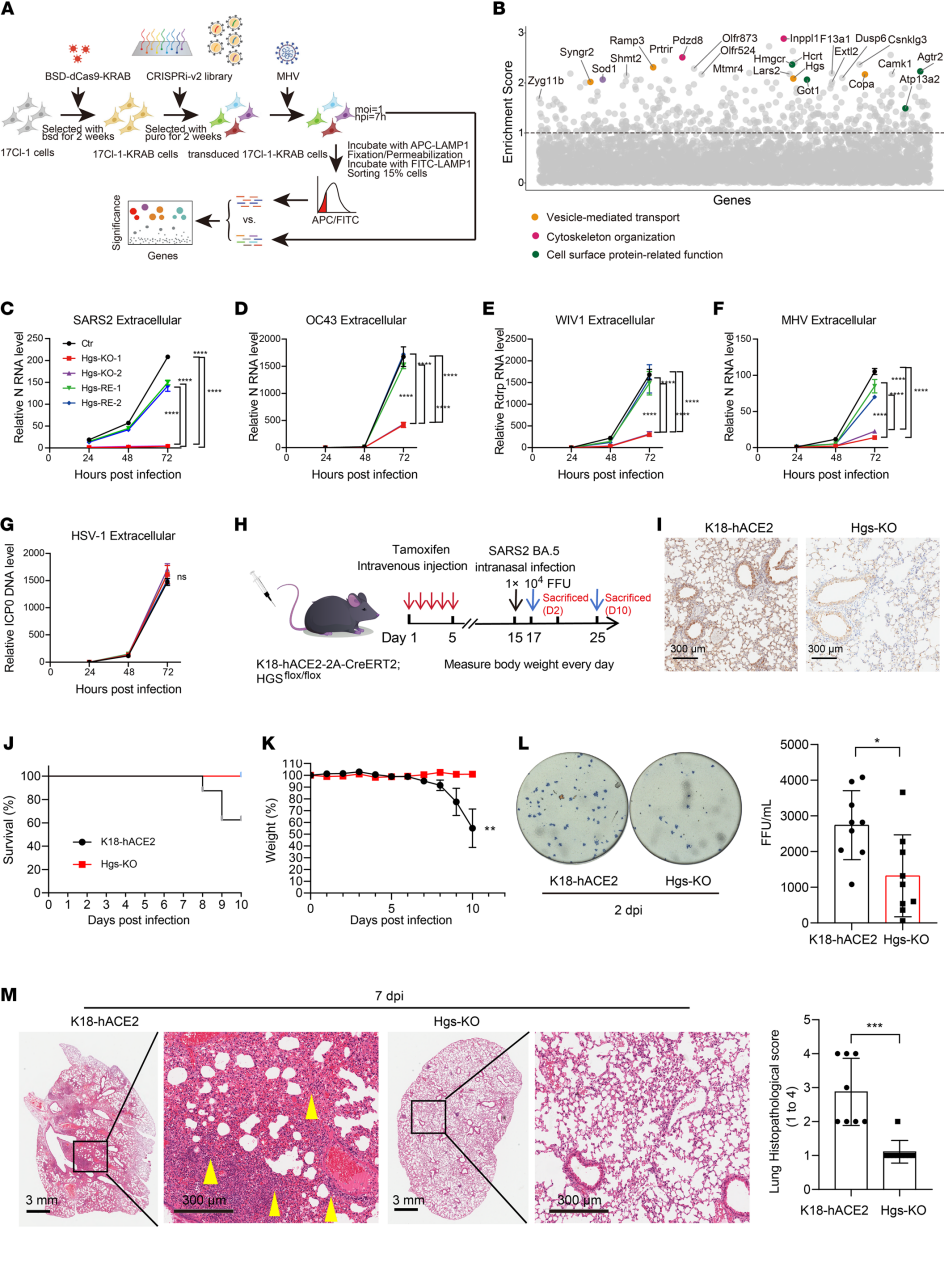
Genome-wide CRISPRi screens identify HGS as a host target for pan-coronavirus therapy. (**A**) Schematic illustrating genome-wide CRISPRi screens based on surface membrane LAMP1 for the identification of host factors for coronavirus assembly and egress. dCas9-KRAB–expressing 17Cl-1 cells transduced with a genome-wide sgRNA library were infected with MHV for 7 h (MOI = 1). The 15% of cells with low ratio of cell surface LAMP1/total LAMP1 were enriched, and sgRNA abundance was determined by next-generation sequencing. (**B**) Gene enrichment for CRISPRi screen. Enrichment scores were determined by MaGECK analysis, and genes were colored by biological function. Dotted line indicates log10 (enrichment score) = 1. All genes and enrichment scores can be found in Supplemental Table 1. (**C**–**G**) RT-qPCR analysis of extracellular SARS-CoV-2 (**C**), HCoV-OC43 (**D**), WIV1 (**E**), and MHV (**F**) viral gRNA and HSV-1 (**G**) viral gDNA levels (hpi = 24 h, 48 h, 96 h, respectively; MOI = 1). *N* = 3 independent biological replications. (**H**) Schematic illustrating conditional *Hgs*-KO K18-hACE2 mice infected with SARS-CoV-2. (**I**) Immunohistochemical staining of HGS in lung of ctr or *Hgs*-KO-K18-hACE2 mice. Scale bar = 300 μm. (**J**, **K**, and **M**) Survival curves (**J**), body weight changes (**K**), and histopathology of hematoxylin and eosin–stained (HE-stained) lung tissues on day 7 (**M**). Quantitative analysis of pathological severity scores based on the percentage of affected area in lung tissues. (*N* = 9 for K18-hACE2 group and *N* = 8 for *Hgs*-KO group.) (**L**) Viral titration by focus-forming assay (FFA) with the supernatant of homogenized lung tissues on day 2 (*N* = 9 for each group). FFU, focus-forming units. Data are the mean ± SD. Significance testing for **C**–**G** was performed with 1-way ANOVA and Tukey’s multiple-comparison test. Significance testing for **K**–**M** was performed with a 2-tailed *t* test. *P ≤ 0.05, **P ≤ 0.005, ***P ≤ 0.0005, ****P ≤ 0.0001.

Having established a suitable cellular model and FACS-based assay for indicating coronavirus egress, we performed a genome-wide CRISPRi screen to identify the key host factors for coronavirus infection (Figure 1A). A total of 434 candidate genes were substantially enriched and classified, which are related to vesicle-mediated transport and cytoskeleton (Figure 1B and Supplemental Table 1). Further validation of the top 24 enriched genes revealed that knockdown of most candidate genes led to a notable decrease in extracellular viral gRNA, while having no effect on or, in some cases, even increasing intracellular viral gRNA levels at 16 hours postinfection (hpi) (Supplemental Figure 1, C and D). This pattern suggests that these candidate genes influence coronavirus assembly and egress, rather than replication, thereby affirming the specificity of the assay. Meanwhile, silencing the subunit of ESCRT, HGS, has significantly reduced coronavirus assembly and egress. Nevertheless, little is known about the role of HGS in coronavirus assembly and egress.

To investigate the role of HGS in the coronavirus infection, *Hgs*-knockout (*Hgs*-KO) Huh7.5.1 and 17Cl-1 cell lines generated using CRISPR/Cas9 were subjected to various coronaviruses, including SARS-CoV-2, HCoV-OC43, bat coronavirus WIV1, and MHV. Quantitative real-time PCR (RT-qPCR) results showed that extracellular viral gRNA levels were dramatically decreased in *Hgs*-KO cells but restored in *Hgs*-rescued cells at 72 hpi (Figure 1, C–F). Notably, extracellular viral gDNA levels were unchanged in *Hgs*-KO and rescued cells following infection with the DNA virus HSV-1 (Figure 1G). These results indicate that HGS functions as a critical host factor for pan-coronavirus infection.

We further investigated the role of HGS in coronavirus infection in vivo. Due to the embryonic lethality of global *Hgs*-KO mice (13), we employed a conditional gene-targeting approach to generate mice with selective HGS deficiency in human angiotensin-converting enzyme 2–expressing (hACE2-expressing) cells (Figure 1, H and I). Strikingly, HGS deficiency markedly attenuated body weight loss and significantly enhanced the survival rate of SARS-CoV-2 Omicron BA.5–infected mice (Figure 1, J and K). Furthermore, HGS deficiency reduced viral titers in the lungs on day 2 postinfection (Figure 1L). Histopathological analysis further revealed that lung inflammation was alleviated in HGS-deficient mice (Figure 1M). These results indicate that HGS functions as a critical host factor for SARS-CoV-2 infection in vivo.

In addition, we selectively inactivated HGS in the liver by intravenously injecting liver-tropic adeno-associated virus (AAV) containing sgRNA specifically targeting the *Hgs* gene into spCas9-knockin mice (Supplemental Figure 2A). Immunoblotting (IB) results revealed a significant decrease in *Hgs* expression in the liver, while no significant change was observed in the lung, as compared with control mice treated with scrambled sgRNA (Supplemental Figure 2, B and C), which confirmed the tissue-specific tropism of the AAV. Subsequently, the mice were intranasally infected with MHV for 5 days. Body weight loss did not show significant differences in the liver-specific *Hgs*-silencing mice compared with control mice (Supplemental Figure 2D). However, viral gRNA levels and virus titers were significantly decreased in the liver but not the lung of the liver-specific *Hgs*-silencing mice (Supplemental Figure 2, E–G). Histological analysis further indicated that liver damage was alleviated in the liver-specific *Hgs*-silencing mice, while acute tissue damage in the lung was not significantly improved (Supplemental Figure 2, H and I). These results collectively demonstrate the important role of HGS in MHV infection in vivo.

### HGS controls pan-coronavirus assembly.

We further investigated whether HGS plays a role in the virus genome replication or virion assembly and egress. HGS deficiency resulted in a significant decrease in secreted viral gRNA levels, while intracellular viral gRNA levels remained unaffected after various coronaviruses’ infection for 16 hours, including MHV, SARS-CoV-2, HCoV-229E, HCoV-OC43, HCoV-NL63, and bat coronavirus WIV1 (Supplemental Figure 3). Conversely, the rescued expression of HGS in the *Hgs-*KO cells restored secreted viral gRNA levels without altering intracellular viral gRNA levels (Supplemental Figure 3). Furthermore, the intracellular protein level of viral structural protein N was unchanged in both *Hgs*-KO and rescued cells (Supplemental Figure 3). These findings strongly indicate that HGS plays a regulatory role in virion assembly and egress, rather than genome replication.

**Figure 2:**
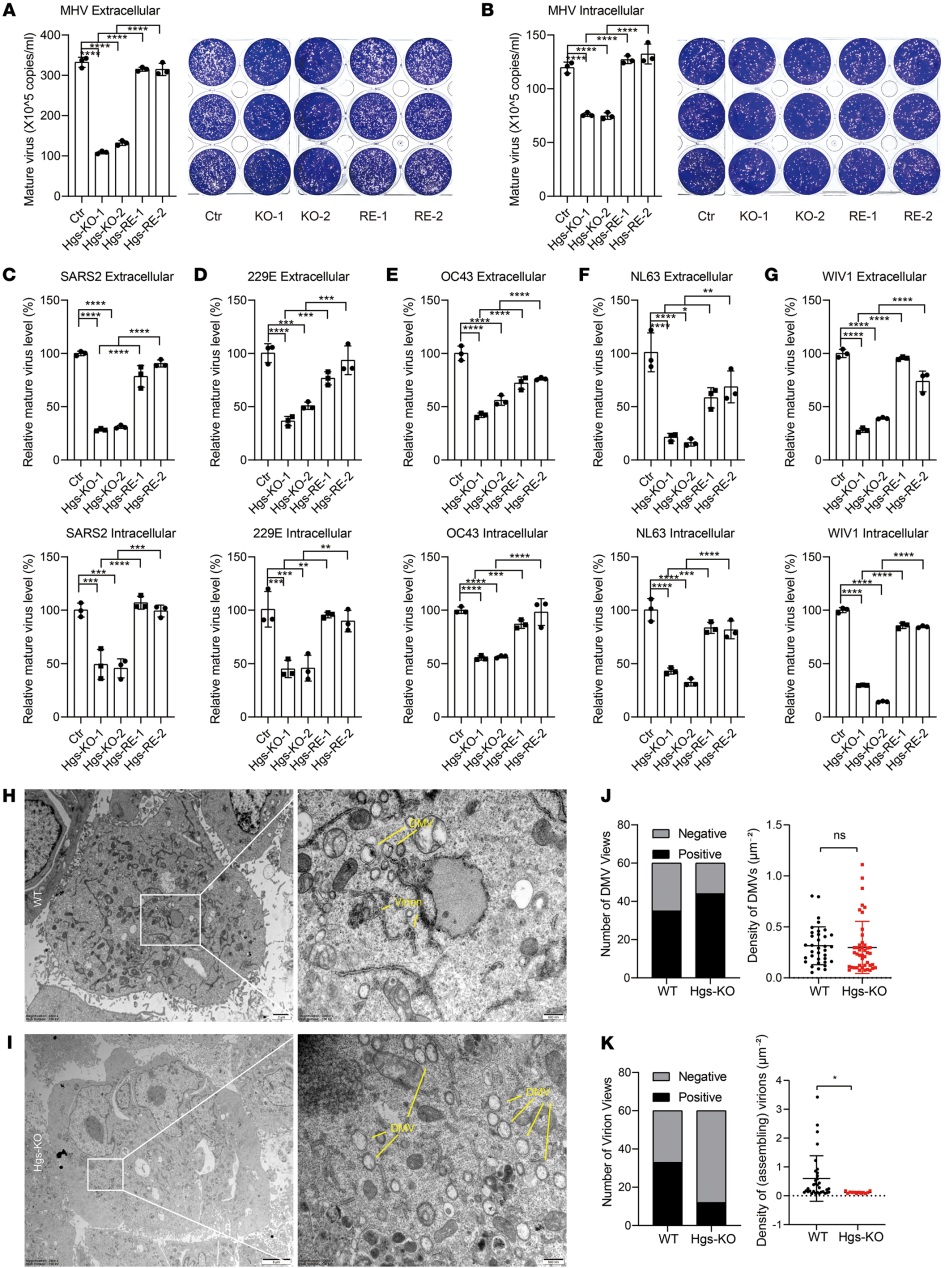
HGS facilitates virion assembly. (**A** and **B**) Plaque assay analysis of extracellular (**A**) and intracellular (**B**) mature MHV titer levels in the Ctr and 2 *Hgs*-KO clones and their respective *Hgs*-rescued 17Cl-1 cells (hpi = 16 h, MOI = 1). *N* = 3 independent biological replications. (**C**–**G**) RT-qPCR analysis of extracellular (upper panel) and intracellular (lower panel) mature virion of SARS-CoV-2 (**C**), HCoV-OC43 (**D**), HCoV-229E (**E**), HCoV-NL63 (**F**), and WIV1 (**G**) in the Ctr and 2 *Hgs*-KO clones and their respective *Hgs*-rescued Huh7.5.1 cells (hpi = 24 h, MOI = 1). The extracellular and intracellular mature virion from Ctr and 2 *Hgs*-KO clones and their respective *Hgs*-rescued Huh7.5.1 cells were subjected to infect WT Huh7.5.1 cells for 6 h. The intracellular viral gRNA levels were examined to indicate the mature virion level. *N* = 3 independent biological replications. (**H**–**K**) TEM analysis of MHV-infected Ctr (**H**) and *Hgs*-KO (**I**) 17Cl-1 cells (hpi = 24 h, MOI = 1). Scale bar, 2 μm (**H** left), 500 nm (**H** right), 5 μm (**I** left), 500 nm (**I** right). (**J**) Quantitative analysis of the number of DMV-positive views (left panel) and the number of DMVs per unit cytoplasmic area (right panel). (**K**) Quantitative analysis of the number of virion-positive views (left panel) and the number of vesicle-contained virions per unit cytoplasmic area (right panel). Data are the mean ± SD. Significance testing for **A**–**G** was performed with 1-way ANOVA and Tukey’s multiple-comparison test. Significance testing for **J** and **K** was performed with a 2-tailed *t* test. *P ≤ 0.05, **P ≤ 0.005, ***P ≤ 0.0005, ****P ≤ 0.0001.

To investigate the role of HGS in regulating coronavirus assembly or release, intracellular and extracellular virus titers were assessed using a virus plaque assay. The results showed a significant reduction in both intracellular and extracellular virus titers in *Hgs*-KO 17Cl-1 cells infected with MHV and an increase in both intracellular and extracellular virus titers in the *Hgs*-rescued cells (Figure 2, A and B), suggesting that HGS predominantly influences coronavirus assembly. Consistently, HGS deficiency also decreased other coronaviruses’ assembly, including SARS-CoV-2, HCoV-229E, HCoV-OC43, HCoV-NL63, and WIV1 (Figure 2, C–G). Furthermore, transmission electron microscope (TEM) observations revealed intracellular mature virions were significantly decreased in *Hgs*-KO cells compared with control cells, while double-membrane vesicles (DMVs), the replication organelles, were not significantly changed (Figure 2, H–K), suggesting that the absence of HGS does not affect replication but impedes virion assembly.

In addition, various coronavirus virus-like particles (VLPs) containing M/envelope small membrane (E)/nucleoprotein (N) proteins were utilized to investigate coronavirus assembly, including SARS-CoV-2, MERS, HCoV-OC43, and HCoV-HKU1. Overexpression of HGS significantly increased the ratio of secreted to intracellular N protein (Supplemental Figure 4, A–D). In contrast, HGS deficiency markedly reduced this ratio, while HGS rescue restored it to levels comparable to those observed in control cells (Supplemental Figure 4, E–H). All these results strongly support the role of HGS in regulating pan-coronavirus assembly.

### HGS interacts with M protein and facilitates its trafficking to ERGIC for virion assembly.

**Figure 3:**
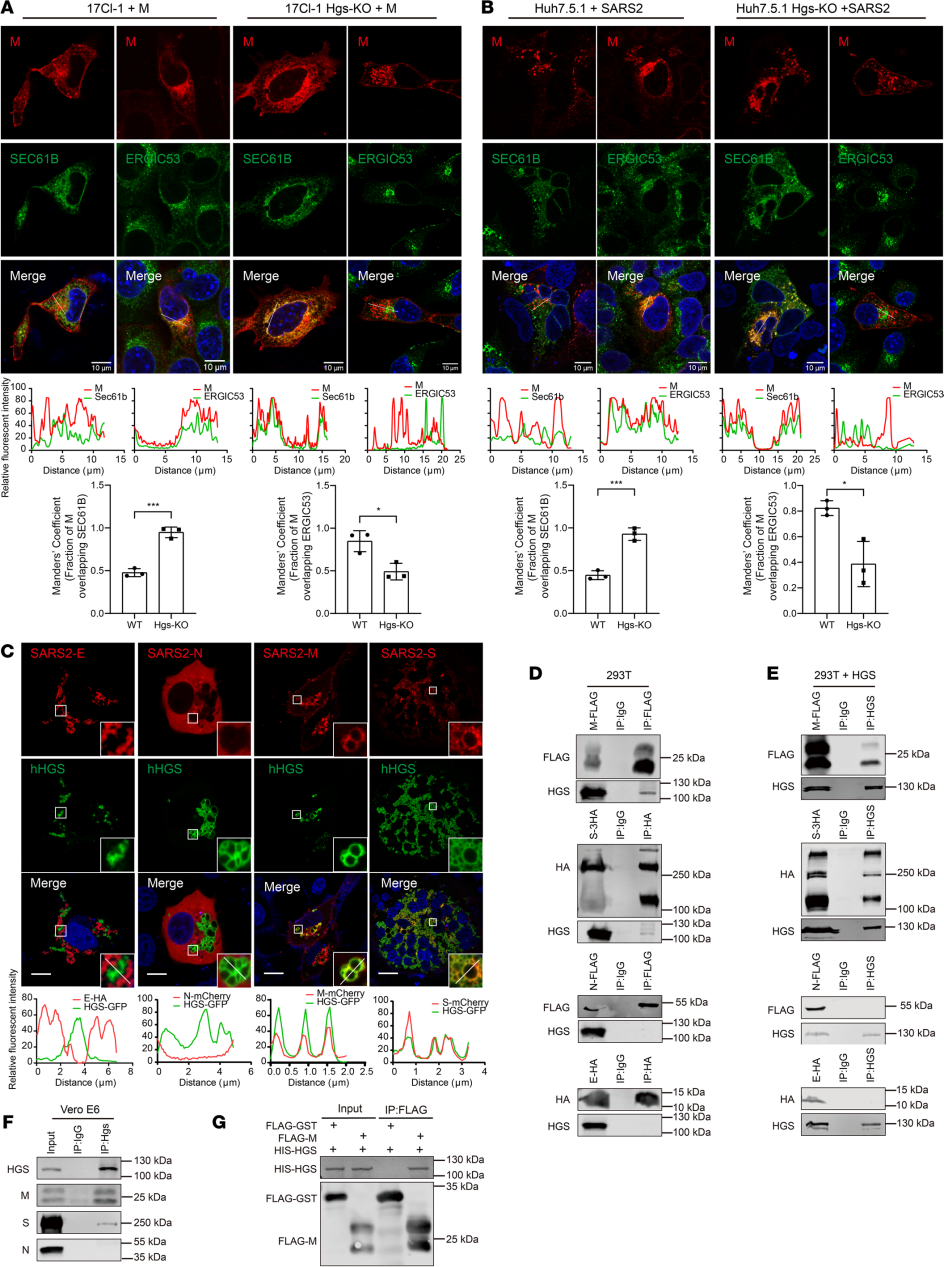
HGS interacts with M protein and facilitates its trafficking to ERGIC. (**A** and **B**) Representative IF analysis of the colocalization of SARS-CoV-2 M with SEC61B or ERGIC53 in WT and *Hgs*-KO 17Cl-1 cells (**A**) or in SARS-CoV2 Omicron BA.5-infected Huh7.5.1 WT and *Hgs*-KO cells (MOI = 0.5) (**B**). Trace outline is used for line-scan analysis of the relative fluorescence intensity of SARS-CoV-2 M with SEC61B and ERGIC53. Scale bar, 10 μm. Quantitative image analysis of M-SEC61B/ERGIC53 colocalization using Manders’ coefficient. *N* = 3 independent biological replications. (**C**) Representative IF analysis of the colocalization of HGS with SARS-CoV-2 M, S, E, and N in Vero E6 cells. Trace outline is used for line-scan analysis of the relative fluorescence intensity of HGS with SARS-CoV-2 M, S, E, and N. Scale bar, 10 μm. *N* = 3 independent biological replications. (**D**–**F**) Co-IP analysis of interaction of individually transiently expressed SARS-CoV-2 M, S, E, N with HGS in HEK293T cells (**D** and **E**) and HGS with M, S, N in SARS-CoV-2–infected Vero-E6 cells (hpi = 24 h) (**F**). Immunoprecipitates pulled down by FLAG/HA (**D**) or HGS (**E** and **F**) antibody were analyzed by IB with indicated antibodies. IgG IP was used as negative control. Input represents 5% of the total cell extract. Molecular weights are in kDa. *N* = 3 independent biological replications. (**G**) In vitro pull-down analysis of the interaction between HGS and SARS-CoV-2 M. Purified HIS-HGS and FLAG-M proteins subjected to pull-down assay by FLAG antibody were analyzed by IB with indicated antibodies. FLAG-GST protein was used as a negative control. Input represents 5% of the total proteins used for pull-down. Molecular weights are in kDa. *N* = 3 independent biological replications. Data are the mean ± SD. Significance testing for **A** and **B** was performed with a 2-tailed *t* test. *P ≤ 0.05, ***P ≤ 0.0005.

We went on to investigate the mechanism by which HGS regulates virion assembly. Previous studies have established that HGS interacts with multiple cellular proteins and modulates their intracellular trafficking and sorting (14, 15). Given that the coronavirus M protein serves as the central organizer of virion assembly, with its proper subcellular localization being important for this process (16–18), we hypothesized that HGS might regulate M protein trafficking to facilitate viral particle formation. In wild-type (WT) cells, M protein was colocalized with an ERGIC marker, consistent with previous studies demonstrating that coronavirus assembly occurs in the ERGIC (19, 20). However, in *Hgs*-KO cells, the M protein colocalized with an ER marker but not with an ERGIC marker (Figure 3A). Consistently, similar results were observed in SARS-CoV-2–infected cells (Figure 3B). Moreover, Brefeldin A (BFA), a known inhibitor of ER-to-Golgi trafficking (21, 22), was used to monitor the ER export of M protein (Supplemental Figure 5, A and B). This kinetic process was effectively blocked by BFA treatment, and importantly, the trafficking was restored after a 5-hour BFA washout in WT cells. However, HGS deficiency blocked the M protein in the ER regardless of BFA treatment. Taken together, these results indicate that HGS facilitates M protein trafficking to ERGIC for virion assembly.

We further explored how HGS facilitates M protein trafficking to ERGIC. Coexpressing HGS with individual SARS-CoV-2 viral structural protein M, E, N, and spike glycoprotein (S) showed that HGS colocalized with viral structural protein M and S but not with E and N (Figure 3C). Subsequent co-immunoprecipitation (co-IP) results showed that HGS was immunoprecipitated by viral structural proteins M and S, not by E and N (Figure 3D). Conversely, viral structural proteins M and S, but not E and N, were immunoprecipitated by HGS (Figure 3E). These results showed that HGS interacted with viral structural proteins M and S, which were further confirmed by endogenous HGS IP in SARS-CoV-2–infected cells (Figure 3F). Moreover, purified HGS protein and M protein were subjected to pull-down assay. IB results indicated interaction between M protein and HGS, as HGS protein was pulled down by M protein but not by the glutathione-*S*-transferase (GST) control protein (Figure 3G). Taken together, all these results showed that HGS interacts with viral structural protein M.

Subsequently, we investigated whether HGS could interact with other coronavirus M proteins. Immunofluorescence (IF) analysis revealed that HGS colocalized with all coronavirus M proteins, including those of MHV, HCoV-229E, HCoV-OC43, HCoV-NL63, HCoV-HKU1, MERS, SARS-CoV-1, and WIV1 (Supplemental Figure 5C). Additionally, different HGS species were observed to colocalize with SARS-CoV-2 M protein (Supplemental Figure 5D). These findings collectively suggest that the interaction between HGS and M protein is conserved across diverse coronaviruses.

To identify the specific domain within HGS required for the interaction with M, various truncations of HGS were generated, including HGS 1-166, HGS 1-215, HGS 1-290, HGS 1-390, and HGS 1-509 (Supplemental Figure 6A). IF analysis showed that HGS 1-290, HGS 1-390, and HGS 1-509 were capable of forming vesicular structures and colocalized with M protein, similar to WT HGS (Supplemental Figure 6B). Consistently, co-IP results showed that HGS 1-290, HGS 1-390, and HGS 1-509, but not HGS 1-166 and HGS 1-215, were immunoprecipitated by M protein (Supplemental Figure 6C). These results demonstrated the important role of the HGS 1-290 region in mediating its interaction with M protein.

To identify the specific domain within M required for the interaction with HGS, various truncations of M protein were generated, including a transmembrane domain truncation 1-118 and an intravirion domain truncation Δ19-100 (Supplemental Figure 6D). IF results showed that HGS colocalized with M intravirion domain truncation Δ19-100 but not with M transmembrane domain truncation 1-118 (Supplemental Figure 6E). Furthermore, the purified HGS protein was pulled down by the M intravirion protein but not by the FLAG control protein (Supplemental Figure 6F), indicating an interaction between HGS and the M intravirion domain. Subsequently, we sought to further narrow the specific binding domain within the M intravirion domain with HGS. The M intravirion domain comprises 8 β-sheet domains, and we sequentially generated 4 truncations that deleted these domains: M Δ118-134, M Δ135-161, M Δ162-189, and M Δ190-204 (Supplemental Figure 6D). IF results revealed that only M Δ135-161 did not colocalize with HGS (Supplemental Figure 6E), indicating the important role of the M 135-161 domain in its interaction with HGS. Additionally, both M 135-161-mCherry and M 135-146-mCherry showed complete colocalization with HGS (Supplemental Figure 6E), further confirming an interaction between the M 135-146 domain and HGS. Collectively, these results show HGS interacts with M protein and facilitates its trafficking to ERGIC for virion assembly.

### M-derived peptides targeting HGS alleviate the coronavirus infection in vitro, in ALI-cultured HBEs, and in vivo.

These pioneering results prompted us to investigate whether the M binding domain could serve as a dominant-negative truncation to disrupt the interaction between HGS and M, thereby preventing coronavirus assembly. To verify this hypothesis, we observed a significant decrease in extracellular virus titer in SARS-CoV-2 M 135-161– and M 135-146–overexpressing 17Cl-1 cells infected with MHV compared with FLAG control cells (Supplemental Figure 7A), indicating that M-derived peptides may impede the HGS-M interaction and hinder coronavirus infection. To expand on the clinical applicability, the M 135-161 and M 135-146 peptides were designed to fuse with HIV-TAT, a cell-penetrating peptide known to facilitate cellular uptake, which we called M161 and M146 here, respectively. Surface plasmon resonance (SPR) results showed that the binding affinity between M146 and M161 with HGS reached to 5.73 nM and 3.84 nM, respectively (Supplemental Figure 7, B and C). The M146 and M161 peptides could significantly block the interaction between HGS and M protein (Supplemental Figure 7D). Cell viability assessments demonstrated that M146 and M161 exhibited no significant cytotoxicity up to 10^4^ nM (Supplemental Figure 7, E–G). Strikingly, M146 and M161 treatment significantly decreased the extracellular viral gRNA levels but not the intracellular viral gRNA levels in a dose-dependent manner after infection with MHV for 24 hours. However, no significant change of extracellular and intracellular viral gRNA level was observed in the control peptide (Supplemental Figure 7, H and I). When coronavirus infection continued for 72 hours, M146 and M161 treatment at 10^4^ nM strikingly decreased the MHV infection but not the HSV-1 virus infection (Supplemental Figure 7, J and K). Furthermore, when M146 and M161 were mutated to decrease their binding affinity to HGS, the inhibition of virus release was consistently reduced (Supplemental Figure 7, L–P). Since HGS is a key host factor for pan-coronavirus assembly, we extended our analysis to various coronaviruses, including SARS-CoV-2, HCoV-NL63, HCoV-229E, HCoV-OC43, and WIV1. Treatment with M146 and M161 at 10^4^ nM for 24 hours markedly decreased extracellular viral gRNA levels across all coronavirus-infected cells, while intracellular viral gRNA levels remained unchanged (Supplemental Figure 7, Q–U), suggesting that targeting HGS could effectively mitigate coronaviruses’ assembly.

The airway ciliated epithelial cells are the main targets for initial coronavirus infections, and spread of progeny to neighboring cells is vital for coronavirus infection (23). Therefore, we differentiated primary HBE cells in ALI cultures to form a bronchial epithelial barrier containing ciliated, goblet, and basal cells (Supplemental Figure 8, A and B). The fully differentiated ALI-cultured HBEs treated with M146 and M161 were inoculated with various coronaviruses, including SARS-CoV-2, HCoV-229E, HCoV-OC43, and HCoV-NL63. IF analysis showed that N proteins were seen most in ciliated cells (Supplemental Figure 8C), consistent with previous results (23). Strikingly, M146 and M161 treatment significantly decreased the N protein levels (Supplemental Figure 8, D–G). Consistently, RT-qPCR analysis of the mucus layer revealed that the viral gRNA was dramatically decreased after treatment with M146 and M161, compared with the FLAG control (Supplemental Figure 8, H–K). Taken together, targeting HGS could effectively mitigate coronavirus infections in ALI-cultured HBEs.

To determine the function of HGS-targeted peptides in vivo, peptide M146 and M161 were modified with d-retroinverso (DRI) isoform to enhance the peptide potency. Several DRI-modified peptides have been reported to be well tolerated and therapeutically effective in clinical trials (24–26). The mice preinjected with M146-DRI and M161-DRI were infected with MHV. Tissue distribution analysis showed that the M146-DRI peptide mainly localized in the lung after intravenous injection (Supplemental Figure 8M). RT-qPCR and virus plaque analysis showed that the viral gRNA and virus titer in the lung were significantly decreased after treatment with M146-DRI and M161-DRI (Supplemental Figure 8, L–P). Consistently, histological analysis indicated that the lung injury was alleviated in the mice treated with M146-DRI and M161-DRI (Supplemental Figure 8Q). Together, these results indicate that targeting HGS could alleviate the coronavirus infection in vivo.

### Small-molecule screening reveals RTB disrupts HGS-M interaction.

**Figure 4:**
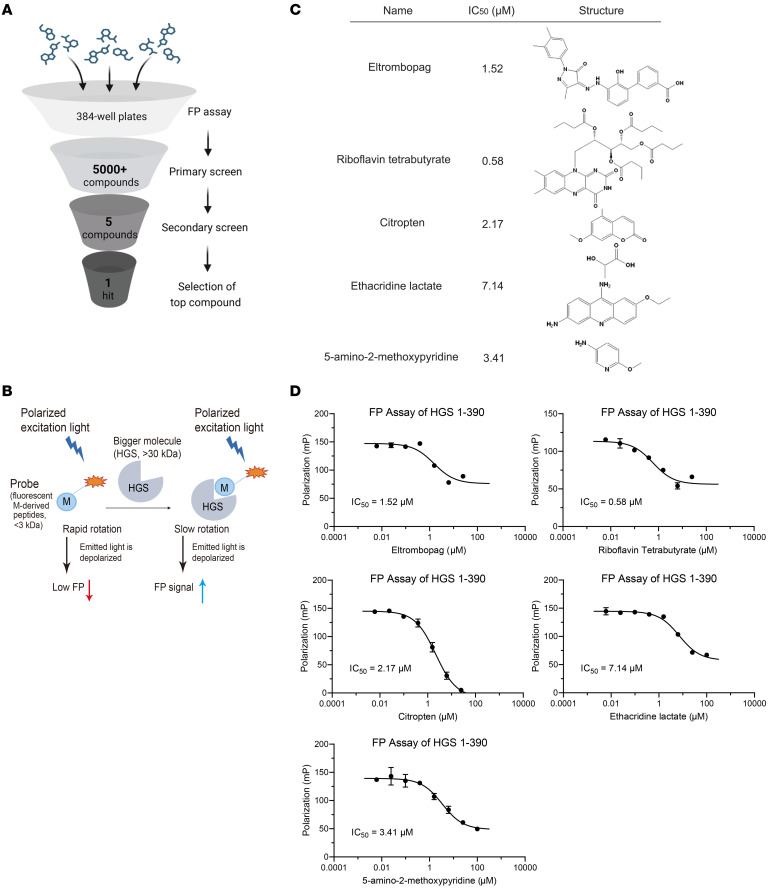
Discovery of a small-molecule inhibitor targeting HGS. (**A**) The flowchart of FP-based high-throughput screening (HTS) assay. (**B**) Schematic representation of the FP-based HTS assay. (**C**) Half of inhibition concentration (IC_50_) and structure of 5 compounds, which showed the best inhibitory activity among all the compounds. (**D**) The IC_50_ values of the top 5 hits were shown in the inhibition curves of HGS 1-390. Data are the mean ± SD and analyzed in GraphPad Prism 9.3.

To identify the small molecules that disrupt the interaction between HGS and M protein, the workflow of compound screening based on fluorescence polarization (FP) assay was applied in a preliminary screening of an in-house drug library of over 5,000 compounds with known structure (Figure 4, A and B). By selecting compounds exhibiting more than 60% inhibition, a list of 5 candidates (eltrombopag, RTB, citropten, ethacridine lactate, and 5-amino-2-methoxypyridine) was identified (Figure 4C). We further measured the IC_50_ values of the hit compounds identified from high-throughput screening, among which RTB exhibited the most potent inhibitory activity (IC_50_ = 0.58 μM) (Figure 4D). Cell viability assays confirmed that all 5 compounds exhibited minimal cytotoxicity (50% cytotoxic concentration [CC_50_] > 20 μM). Among these, RTB most effectively rescued coronavirus-induced cytopathic effects, with an EC_50_ of 3.48 μM. This antiviral potency was benchmarked against remdesivir (RDV; EC_50_ = 0.69 μM) (Supplemental Figure 9). This pilot screening further illustrated that the FP-based assay can be used as a robust HTS assay for the discovery of HGS protein inhibitors.

### RTB exhibits anti-coronavirus activity in vitro, in ALI-cultured HBEs, and in vivo.

**Figure 5:**
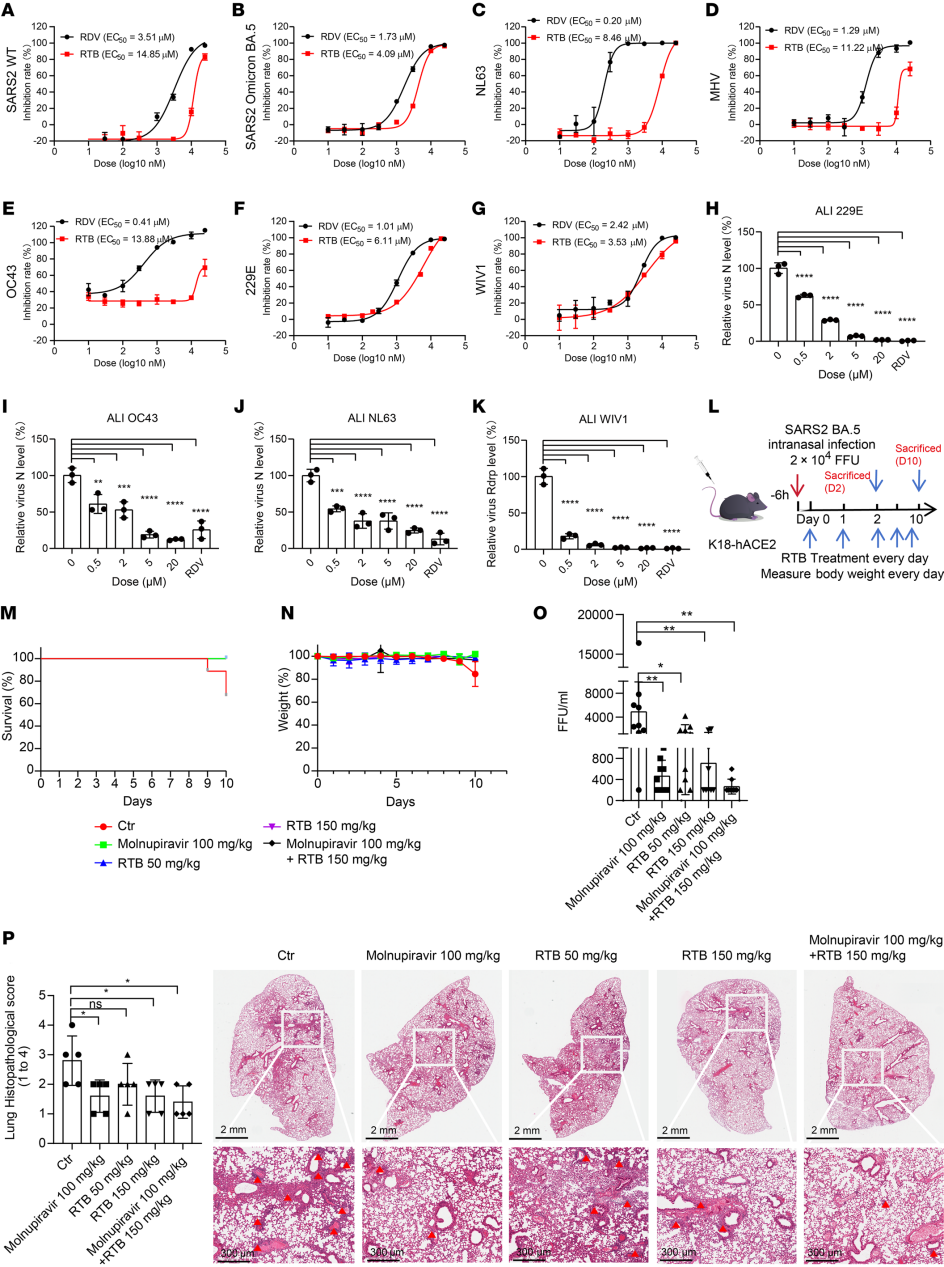
RTB exhibits anti-coronavirus activity in vitro, in ALI-cultured HBEs, and in vivo. (**A**–**G**) Dose titration of RTB and RDV for anti-coronavirus activity. Various concentrations of RTB and RDV were used to assess ability to inhibit various coronaviruses’ infection by indirect immunofluorescence assay (IFA). EC_50_ values of antiviral ability are indicated. (**A**) SARS-CoV-2 wild-type (MOI = 0.1, hpi = 24 h), (**B**) SARS-CoV-2 Omicron BA.5 (MOI = 0.1, hpi =24 h), (**C**) HCoV-NL63 (MOI = 0.1, hpi = 48 h), (**D**) MHV (MOI = 0.1, hpi = 24 h), (**E**) HCoV-OC43 (MOI = 0.1, hpi = 48 h), (**F**) HCoV-229E (MOI = 0.1, hpi = 48 h), (**G**) WIV1 (MOI = 0.1, hpi = 48 h). (**H**–**K**) RTB alleviates coronavirus infection in ALI-cultured HBEs. RT-qPCR analysis of extracellular viral gRNA levels in HCoV-229E (MOI = 1) (**H**), HCoV-OC43 (MOI = 1) (**I**), HCoV-NL63 (MOI = 1) (**J**), WIV1 (MOI = 1) (**K**) –infected ALI-cultured HBEs with treatment with different doses of RTB or 5 μM RDV for 96 h. (**L**) Schematic illustrating SARS-CoV-2 Omicron BA.5 infection in K18-hACE2 mice with treatment with or without RTB. SARS-CoV-2 Omicron BA.5-infected mice (2 × 10^4^ FFU) were treated with PBS, molnupiravir (100 mg/kg body weight), RTB (50 or 150 mg/kg body weight), or molnupiravir (100 mg/kg body weight) with RTB (150 mg/kg body weight) every 24 h for 2 or 10 days. (**M** and **N**) Survival curves (**M**) and body weight changes (**N**) were analyzed after infection with SARS-CoV-2 Omicron BA.5 (*N* = 9 for each group). (**O**) Viral titration by FFA with the supernatant of homogenized lung tissues on day 2 (*N* = 9 for each group). (**P**) Histopathology of formalin-fixed and HE-stained lung tissues on day 2. Quantitative analysis of pathological severity scores based on the percentage of affected area in lung tissues (*N* = 5 for each group). Data are the mean ± SD. (**H**–**K**, **O**, and **P**) One-way ANOVA and Tukey’s multiple-comparison test. *P ≤ 0.05, **P ≤ 0.005, ***P ≤ 0.0005, ****P ≤ 0.0001.

We further explored the role of RTB in the various coronaviruses’ infection. RTB demonstrated broad-spectrum anti-coronaviral infection with the following EC_50_ values: 14.85 μM for SARS-CoV-2 WT, 4.09 μM for SARS-CoV-2 Omicron BA.5, 8.46 μM for HCoV-NL63, 11.22 μM for MHV, 13.88 μM for HCoV-OC43, 6.11 μM for HCoV-229E, and 3.53 μM for WIV1, respectively (Figure 5, A–G). Notably, all EC_50_ values for RTB were higher than those of RDV. In fully differentiated ALI cultures of HBEs, RTB produced significant, dose-dependent inhibition of HCoV-OC43, HCoV-229E, HCoV-NL63, and WIV1 infection (Figure 5, H–K).

To determine the function of RTB in vivo, K18-hACE2 C57BL/6 mice were infected with the SARS-CoV-2 Omicron BA.5 variant with RTB administered at the onset of infection (Figure 3L). RTB treatment significantly improved survival and markedly reduced body weight loss in infected mice (Figure 5, M and N). Consistently, RTB substantially lowered pulmonary viral titers and alleviated lung inflammation (Figure 5, O and P).

To further explore whether RTB could be an effective antiviral against other coronaviruses in vivo, MHV-infected WT C57BL/6 mice and 229E-infected K18-hACE2 C57BL/6 mice were administered RTB at the onset of infection (Supplemental Figure 10). Remarkably, RTB treatment significantly reduced viral gRNA and lowered pulmonary viral titers in both models. Critically, RTB markedly attenuated virus-induced lung injury (Supplemental Figure 10). Collectively, these results suggest that RTB could alleviate various coronaviruses’ infection in vivo.

### RTB directly targets HGS.

**Figure 6:**
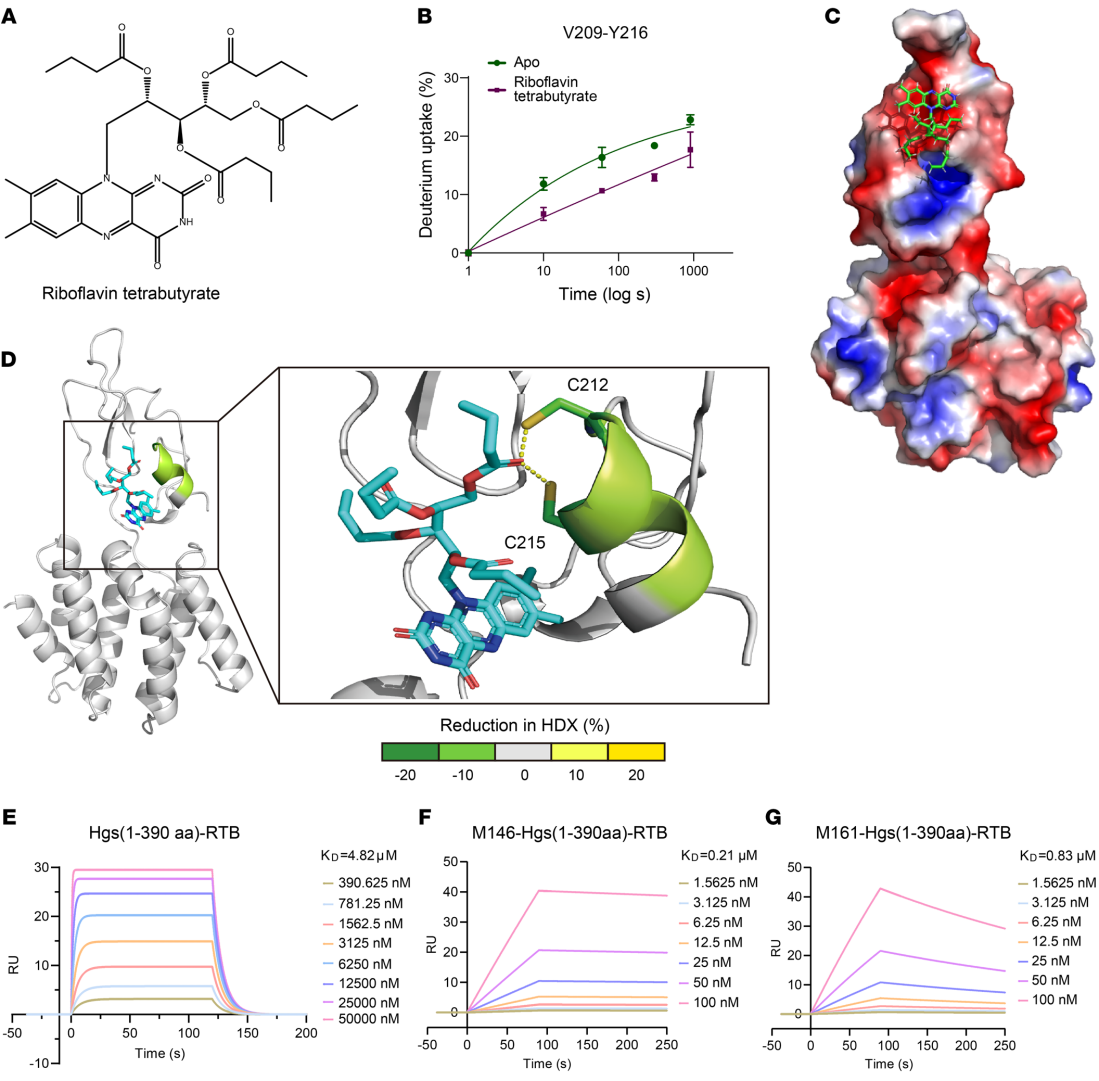
RTB directly targets HGS. (**A**) The chemical structure of RTB. (**B**–**D**) Differential HDX-MS analysis of HGS 1-390 in the presence and absence of RTB is shown as the change in deuterium uptake mapped onto the crystal structure of HGS (PDB: 3ZYQ), highlighting regions affected by RTB binding. The predicted binding pose of RTB with HGS based on the docking result, with RTB represented as indigo sticks (**D**) and surface representation (**C**). Deuterium uptake plots for His-tag HGS affected by RTB binding region (green) in the absence (dark green) or presence of RTB (purple), revealing RTB-induced stabilization effects (**B**). (**E**–**G**) SPR analysis of the binding affinity between HGS 1-390 and RTB (**E**), and binding affinity between HGS 1-390 and peptides M146 (**F**) or M161 (**G**) in the presence of RTB. *N* = 3 independent biological replications. Data are the mean ± SD and analyzed in GraphPad Prism 9.3.

To investigate the conformational change of HGS induced by RTB, we performed hydrogen-deuterium exchange mass spectrometry (HDX-MS), a widely established technique for probing protein structure, dynamics, folding, and interactions. The different deuterium uptake rates of HGS 1-390 upon RTB binding revealed the specific regions that interact with RTB as well as conformational changes induced by RTB binding. Two regions showed significantly different deuterium incorporation in treated HGS with or without RTB. Interestingly, one region, covering residues 167–175, showed an average 6% increase in deuterium uptake, indicating that the binding of RTB to HGS may have altered the conformational change in this region making it more susceptible to exchange with deuterium water. The other region (residues 209–216) exhibited an approximate 10% decrease in deuterium uptake upon HGS binding to RTB, suggesting that this region may be affected by the binding between RTB and HGS (Figure 6, Α–D).

We further explored the direct interaction between HGS and RTB using SPR assay. The results confirmed direct binding between RTB and HGS, with a *K_D_* value of 4.82 μM (Figure 6E). Notably, RTB treatment reduced the binding affinity of HGS for M146 and M161, increasing their *K_D_* values from 5.73 nM and 3.84 nM to 0.21 μM and 0.83 μM, respectively (Figure 6, F and G).

To investigate whether RTB exerts its anti-coronaviral activity through host factor HGS, the *Hgs*-KO cells were treated with RTB or RDV. HGS ablation significantly reduced coronavirus infection (Supplemental Figure 11). Strikingly, RTB treatment in *Hgs*-KO cells failed to inhibit viral infection compared with DMSO-treated *Hgs*-KO cells (Supplemental Figure 11). In contrast, RDV maintained potent antiviral activity in *Hgs*-KO cells (Supplemental Figure 11). These results demonstrate that RTB’s anti-coronaviral mechanism is HGS dependent, whereas RDV acts through an HGS-independent pathway.

### RTB inhibits virion assembly.

**Figure 7:**
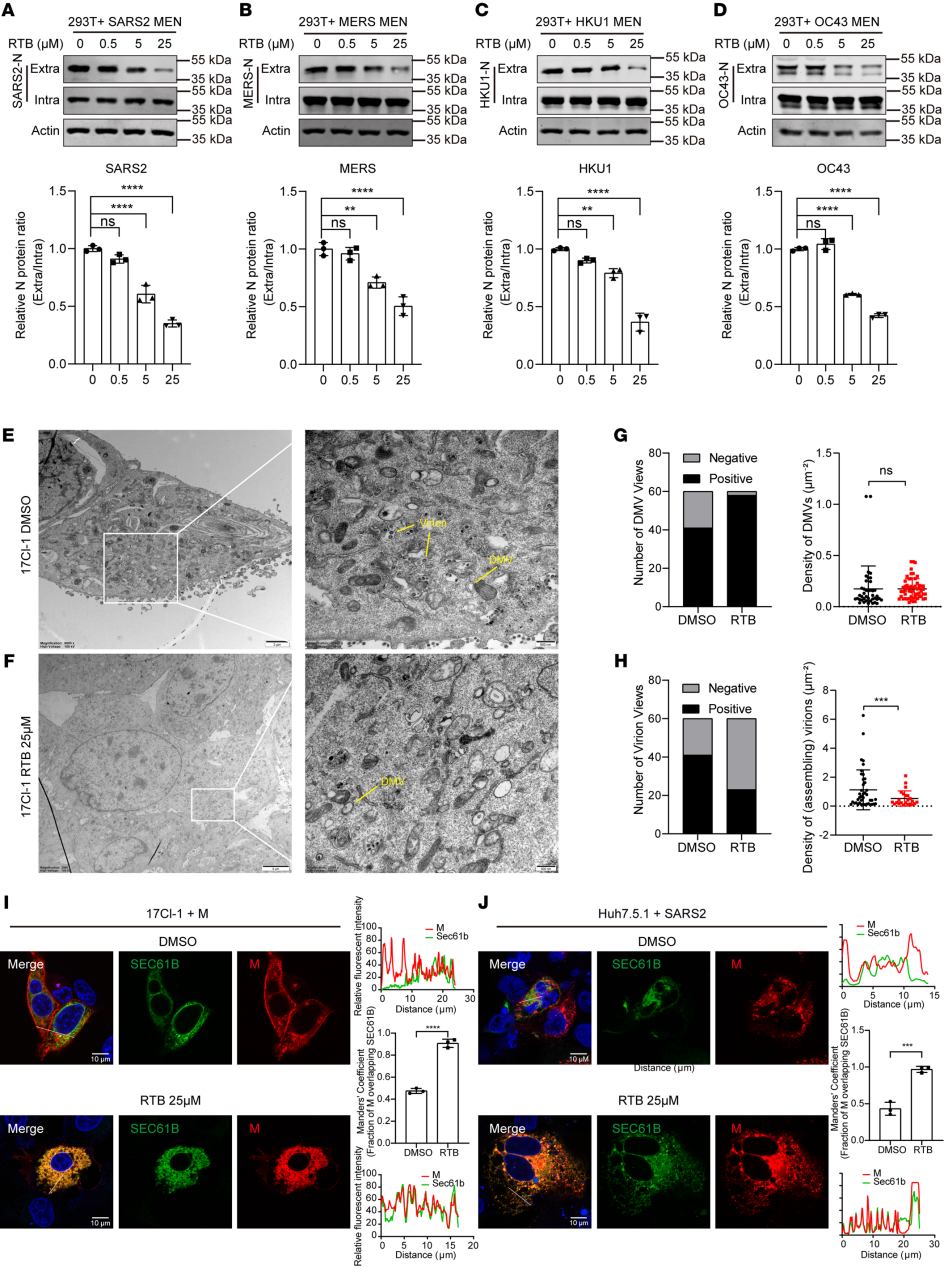
RTB inhibits virion assembly by trapping the M protein in the ER. (**A**–**D**) Representative IB analysis of N protein for indicating SARS-CoV-2 (**A**), MERS (**B**), HCoV-HKU1 (**C**), and HCoV-OC43 (**D**) VLP production in RTB-treated HEK293T cells. HEK293T cells were transfected with VLP system for 48 h. The extracellular secreted protein and intracellular cell lysis were examined by IB with the indicated antibody. Molecular weights are in kDa. *N* = 3 independent biological replicates. (**E**–**H**) TEM analysis of DMSO-treated (**E**) and RTB-treated (25 μM) (**F**) MHV-infected 17Cl-1 cells (hpi = 24 h, MOI = 1). (**G**) Quantitative analysis of the number of DMV-positive views (left panel) and the number of DMVs per unit cytoplasmic area (right panel). (**H**) Quantitative analysis of the number of virion-positive views (left panel) and number of vesicle-contained virions per unit cytoplasmic area (right panel). (**I**) Representative IF analysis of the colocalization between SEC61B and SARS-CoV-2 M-mCherry protein in DMSO- and RTB-treated (25 μM) 17Cl-1 cells. Scale bar, 10 μm. Quantitative image analysis of M-SEC61B colocalization using Manders’ coefficient. *N* = 3 independent biological replicates. (**J**) Representative IF analysis of the colocalization between SEC61B and M protein in SARS-CoV-2–infected Huh7.5.1 cells treated with or without RTB (25 μM). Scale bar, 10 μm. Quantitative image analysis of M-SEC61B colocalization using Manders’ coefficient. *N* = 3 independent biological replicates. Data are the mean ± SD. Significance testing for **A**–**D** was performed with 1-way ANOVA and Tukey’s multiple-comparison test. Significance testing for **G**–**J** was performed with a 2-tailed *t* test. **P ≤ 0.005, ***P ≤ 0.0005, ****P ≤ 0.0001.

To investigate whether RTB acts on the virion assembly, the effect of RTB on the formation of various coronavirus VLPs was studied. RTB treatment resulted in a concentration-dependent inhibition of VLPs’ release (Figure 7, A–D). To confirm these results in cells infected with coronavirus, the effect of RTB was investigated by TEM. TEM observations revealed intracellular mature virions were significantly decreased after treatment of RTB compared with DMSO, while DMVs, the replication organelles, were not significantly changed (Figure 7, E–H), suggesting that the RTB impedes virion assembly, not virus replication. Consistent with the effect of HGS deficiency on the M protein localization, the RTB treatment induced M retention in the ER (Figure 7, I and J). Collectively, these results demonstrate that RTB prevents M protein trafficking to ERGIC, thereby inhibiting virion assembly.

Targeted viral assembly inhibitors offer an advantage over polymerase inhibitors in postinfection antiviral therapy and are ideally suited for combination therapies with current protease and polymerase inhibitors. RTB exhibited similar antiviral activity whether treatment began at infection onset or 6 hpi (Supplemental Figure 12A). In contrast, the antiviral activity of the polymerase inhibitor RDV decreased in a time-dependent manner when treatment was initiated postinfection (Supplemental Figure 12B). Furthermore, RTB demonstrated additive effects in vitro when combined with either molnupiravir or nirmatrelvir (Supplemental Figure 12, C and D). RTB combined with molnupiravir also additively decreased viral titers in the lungs of SARS-CoV-2–infected mice (Figure 5O).

## Discussion

In this study, we utilized a genome-wide CRISPRi screen to identify HGS as a critical host target for pan-coronavirus therapy. We found that HGS interacts with M protein, facilitating its trafficking to the ERGIC, a critical step for virion assembly. HGS deficiency or disruption of HGS-M interaction induced M retention in the ER, thereby inhibiting virion assembly. Using an HTS of 5,000+ FDA-approved or commonly used drugs, we discovered small molecules that bind HGS and disrupt its interaction with the M protein. Among these, RTB exhibits anti-coronavirus activity in vitro, in ALI-cultured HBEs, and in vivo. Collectively, our findings establish HGS as a druggable host target and identify RTB as a promising broad-spectrum antiviral candidate. This work underscores host-directed therapy as a viable strategy against coronaviruses and potentially other viruses.

Targeting virion assembly has emerged as a novel approach for antiviral therapy, with ongoing clinical trials (27). For example, core protein allosteric modulators have shown efficacy in inducing the assembly of empty and aberrant particles in hepatitis B virus (28, 29). In the case of HIV-1, peptides derived from the capsid protein have been utilized to hinder polymerization of capsid protein and inhibit virion assembly (30). Subsequently, larger scale screens for small molecules were performed to identify the inhibitors of HIV-1 assembly or maturation (31). Bevirimat, an inhibitor of HIV-1 assembly and maturation, has shown promising results in clinical trials (32–34). Very recently, 2 small molecules directly targeting the coronavirus M protein were screened by different groups as inhibitors of viral infection (17, 18). These molecules bind and stabilize the M protein in its short form, preventing the conformational switch to the long form required for successful virion assembly. However, these compounds exhibited antiviral activity only against a subset of coronaviruses, and resistance emerged in some strains carrying M protein mutations. Consequently, host factors represent promising alternative antiviral targets with potential for broad-spectrum drug development. In this study, we demonstrate that the host factor HGS facilitates pan-coronavirus infection. HGS interacts with the M protein, facilitating its trafficking to the ERGIC for virion assembly. Precise M protein localization is critical for assembly. During ER-to-ERGIC trafficking, the M protein conformation converts from the long form to the short form (16). We therefore propose that HGS deficiency traps M in the ER, stabilizing the short form and preventing virion assembly. Targeting the HGS-M interaction thus constitutes a promising antiviral strategy.

First, we designed HGS-targeting peptides derived from the viral M protein to disrupt the HGS-M interaction. These peptides demonstrated broad-spectrum anti-coronaviral activity in vitro, in ALI-cultured HBEs, and in vivo. This validates viral protein-derived peptides targeting host factors like HGS as a promising antiviral strategy. In the early 1990s, the HIV-1 HR2 domain–derived peptide SJ-2176 could inhibit HIV-1 infection in vitro, leading to the 2003 FDA-approved T20 (Fuzeon, enfuvirtide), the first HIV fusion inhibitor-based anti-HIV peptide drug (35). Surprisingly, an HCoV-OC43 HR2-derived peptide, EK1, targeting the HR1 domains of human coronavirus S proteins, has been confirmed to be effective in inhibiting infection of all circulating human coronaviruses tested, including SARS-CoV, MERS-CoV, HCoV-229E, HCoV-NL63, and HCoV-OC43, as well as 3 bat SARS-related CoVs (36). Our study now establishes a distinct strategy: using viral protein-derived peptides to target important host factors for antiviral activity.

Furthermore, leveraging structure-activity insights from our peptide inhibitor, we developed a screening strategy to identify FDA-approved or commonly used small molecules disrupting the HGS-M interaction. This approach identified the vitamin B_2_ derivative RTB, which demonstrated broad-spectrum anti-coronaviral activity in vitro, in ALI-cultured HBEs, and in vivo. Crucially, RTB treatment induced M protein retention in the ER, phenocopying the effect of HGS deficiency. This demonstrates that disrupting the HGS-M interaction recapitulates the functional consequences of HGS loss. While targeting an important host factor like HGS raises potential cytotoxicity concerns, RTB exhibited no cytotoxicity or adverse effects in mice even at high concentrations (500 mg/kg). Moreover, as a derivative of vitamin B_2_ (riboflavin), RTB has a well-established safety profile from its long-standing use in humans as a dietary supplement. We also provide evidence that RTB directly interacts with HGS and exerts its antiviral effect through this host factor. Although RTB’s antiviral potency is lower than the polymerase inhibitor RDV, it offers distinct advantages: RTB maintains efficacy in postinfection therapy and shows strong potential for combination regimens with current protease and polymerase inhibitors. Future work will focus on determining the structure of the HGS-M complex and developing higher affinity small molecules targeting HGS.

In addition, our study provides a critical resource for studying coronavirus assembly and egress. Previous studies have predominantly focused on using long-term, cytopathic effect–based, genome-wide CRISPR/Cas9 screens to identify key host factors involved in coronavirus entry and replication, with a limitation in identifying factors associated with virion assembly and egress (4–8, 37, 38). This is because inhibiting viral entry and replication can enhance cell survival; however, even if virion assembly and egress are obstructed, the virus can still generate cytotoxic viral proteins and induce endo-membrane rearrangements in host cells. Inhibiting virus assembly and egress only hinders the spread of progeny to neighboring cells without alleviating the cytopathic effect within already-infected cells. As a result, long-term, cytopathic effect–based screens are more likely to identify host factors primarily involved in viral entry and replication rather than in virion assembly and egress. In this study, LAMP1 was introduced as an indicator for coronavirus egress, providing a specific means to identify host factors crucial for virion assembly and egress.

Interestingly, some of these confirmed hits are related to pathways that have also been uncovered in other genome-wide CRISPR screens based on cell survival. Specifically, we found that the rate-limiting enzyme in cholesterol synthesis, HMGCR, is crucial for coronavirus assembly and egress, highlighting the importance of cholesterol homeostasis in virion assembly. Several other genome-wide CRISPR screens based on long-term cytopathic effects have also identified proteins involved in regulating cholesterol homeostasis as key host factors for SARS-CoV-2 and HCoV-OC43 infection (5, 6). Notably, statins, which inhibit HMGCR and thereby reduce cholesterol production, have been associated with improved outcomes in patients with COVID-19 (39, 40). Furthermore, genes involved in the biosynthesis and transport of glycosaminoglycans, such as EXT1, EXT2, and EXTL3, are important host factors for carrying out the pan-coronavirus life cycle (6). This aligns with our findings that EXTL2 plays a role in regulating coronavirus assembly and egress. Additionally, some of the identified hits in this study have been shown to regulate infection by other enveloped viruses. For example, INNPL1 (also known as SHIP2) is involved in poxvirus dissemination (41), PDZD8 binds to the HIV-1 Gag polyprotein and enhances HIV-1 infection efficiency (42), and COPA interacts with and regulates trafficking of the SARS-CoV-2 S protein between the ER and Golgi apparatus (43). Overall, this study offers a valuable resource for investigating host factors that play a role in controlling coronavirus assembly and egress.

HGS is a subunit of the ESCRT, which plays a key role in mediating endosomal vesicle budding and the formation of multivesicular bodies (44). Most enveloped virus families have evolved to utilize ESCRT components, particularly TSG101 and ALIX, to facilitate virus particle assembly and recruit ESCRT-III and VSP4 complexes for terminal scission events (45). Generally, the structural proteins of enveloped viruses contain specific late domains such as P(S/T)AP, PPXY, or YPX_n_L (n≤3) that interact with various ESCRT components to facilitate virus assembly and budding (46, 47). However, coronavirus M protein does not possess these canonical late domains, raising questions about the involvement of ESCRT components in this process. In this study, it was discovered that HGS interacts with and recruits coronavirus M protein to the ERGIC for virion assembly. This is a potentially novel mechanism for virion assembly independent of the late domain signature. Recently, other ESCRT components TSG101 and VPS28 have been demonstrated to play a critical role in membrane budding and terminal scission during coronavirus assembly (48). All these results suggested that ESCRT components participate in coronavirus assembly processes.

## Methods

### Sex as a biological variable

Our study examined male and female mice, and we did not observe significant differences between sexes.

### Cell culture and virus

HEK293T, 17Cl-1, LLC-MK2, Vero E6, Huh7, and Huh7.5.1 cells were cultured in Dulbecco’s modified Eagle medium. HRT-18 cells were cultured in RPMI 1640 medium. Calu3 cells were grown in Eagle minimal essential medium. All cells were cultured in indicated medium supplied with 10% fetal bovine serum and 1% antibiotics at 37°C with 5% CO_2_. A list of cell lines used in this study is provided in Supplemental Table 2.

MHV was a gift from Institute of Biophysics, Chinese Academy of Sciences, Beijing, China. HCoV-OC43 (ATCC VR-1558), HCoV-229E (ATCC VR-740), and HCoV-NL63 (ATCC NR-470) were gifted from Guangzhou National Laboratory, Guangzhou, China. HSV-1 was gifted from Zhongshan School of Medicine, Sun Yat-Sen University, Guangzhou, China. WIV1 was provided by Guangzhou National Laboratory, Guangzhou, China. SARS-CoV-2 (Genbank accession no. MT123290.1) was a clinical isolate obtained from the First Affiliated Hospital of Guangzhou Medical University. MHV, HCoV-OC43, HCoV-229E, HCoV-NL63, and SARS-CoV-2 were propagated in 17Cl-1, HRT-18, Huh-7, LLC-MK2, and Vero E6 cells, respectively. Viral stocks were stored at –80°C. SARS-CoV-2 infection was conducted in Guangzhou Customs District Technology Center BSL-3 Laboratory and Guangzhou National Laboratory BSL-3 Laboratory, while other infections were carried out in BSL-2 laboratory of Guangzhou National Laboratory.

### Genome-wide CRISPRi screen

The genome-wide CRISPRi screen was modified from a previous report (49). 17Cl-1 stably expressing dCAS9-KRAB cells were transduced with lentiviruses carrying the mCRISPRi-v2 library (MOI = 0.3, provided by Likun Wang, Institute of Biophysics, Chinese Academy of Sciences) followed by puromycin selection (2 μg/mL) for 5 days to generate library cells. Subsequently, a total of 1 × 10^9^ library cells were infected with MHV at MOI = 1 for 7 hours. Following infection, the cells were harvested with accutase (STEMCELL Technologies catalog 07920) and stained with APC anti-human CD107a (LAMP-1) antibody (BioLegend catalog 328620). The cells were washed with unbound antibody, fixed with BD Cytofix/Cytoperm solution (catalog 554722), and permeabilized by washing in 1× BD Perm/Wash buffer (catalog 554723). The fixed/permeabilized cells were then suspended in BD Perm/Wash buffer containing FITC anti-human CD107a (LAMP-1) antibody, incubated at 4°C in the dark for 30 minutes, and washed before flow cytometric analysis. Cells were filtered through a 40 μm cell strainer before FACS using a BD FACSAria III. Cell sorting was performed based on the ratio of APC and FITC signals of the cells.

Sorted cells were subjected to extract cellular DNA using the NucleoSpin Blood (MACHEREY-NAGEL catalog 740951.50) according to the manufacturer’s instruction. SgRNA sequences were amplified using Phanta Max Super-Fidelity DNA Polymerase (Vazyme catalog P505-d1), and amplicons were purified from 2.5% agarose gel. Amplicons were sequenced using an Illumina NovaSeq 6000. Critical commercial assays used in this study are provided in Supplemental Table 7. The sgRNA library (CRISPRi_v2_mouse) was prepared as comma-separated file containing siRNA ID, sequence, and the target gene. The raw sequencing reads (FASTQ files) were trimmed to remove adaptors by Cutadapt (ver. 4.6). Mapping, counting, enrichment analysis of sgRNA, and gene-level scores were performed using MAGeCK package (ver.0.5.9.5).

### Virus infection and determination of viral gRNA levels and virus titers

For MHV and HSV-1 infection, viruses were added into the 17Cl-1 or Vero E6 culture medium for 2 hours at MOI = 1, respectively. Culture medium was replaced by fresh complete medium after 2 washes of PBS, and infected cells were maintained at 37°C with 5% CO_2_ for 24 hours, 48 hours, and 72 hours to collect infected cell lysate and supernatant for analysis. For HCoV-OC43, HCoV-229E, and HCoV-NL63 infection in Huh7 or Vero E6, after adsorption at 34°C for 2 hours (MOI = 1), the virus inoculum was removed, and cells were washed with PBS. Medium was replaced and cells were maintained at 34°C with 5% CO_2_ for 24 hours to collect infected cell lysate and supernatant. For SARS-CoV-2 infection in Huh7 or Vero E6, after adsorption at 37°C for 2 hours (MOI = 0.5), the virus inoculum was removed and cells were washed with PBS. Fresh medium was replaced and cells were maintained at 37°C with 5% CO_2_ for 24 hours to collect infected cell lysate and supernatant.

The virus titration was evaluated by RT-qPCR detection of viral N, RdRp, or ICP0 genes. RNA virus MHV, HCoV-OC43, HCoV-229E, HCoV-NL63, and SARS-CoV-2 viral gRNA were extracted from cells or supernatants and reverse-transcribed into cDNA, while DNA virus HSV-1 viral gDNA was extracted from supernatants and analyzed by RT-qPCR. MHV titer was also measured by virus plaque assay.

Briefly, for intracellular mature MHV, HCoV-OC43, HCoV-229E, and HCoV-NL63 titer determination, approximately 1 × 10^6^ infected cells were lysed in 500 μL complete medium by liquid nitrogen freeze-thaw pretreatment. A total of 100 μL medium with mature HCoV-OC43, HCoV-229E, or HCoV-NL63 virus was collected and then added into cell culture to infect 2 × 10^5^ Huh7.5.1 cells for 6 hours. Total RNA was extracted from infected cells using TRIzol (catalog 15596018CN), reverse-transcribed into cDNA using HiScript III 1st Strand cDNA Synthesis Kit (+ gDNA wiper) (catalog R312-02), and analyzed by RT-qPCR. The intracellular mature HCoV-OC43, HCoV-229E, and HCoV-NL63 virus titers were measured and represented as the N gene level in the infected cell.

For extracellular mature HCoV-OC43, HCoV-229E, and HCoV-NL63 virus, 100 μL medium with mature virus was collected and then added into cell culture to infect 2 × 10^5^ cells, while titers were measured and represented in the same way.

For intracellular virus replication detection, total RNA was extracted from cells using TRIzol, reverse-transcribed into cDNA using HiScript III 1st Strand cDNA Synthesis Kit (+gDNA wiper), and analyzed by RT-qPCR according to the manufacturer’s protocol.

For quantification of released extracellular virus, total viral RNA or DNA was isolated from the supernatant, and EasyPure Viral DNA/RNA Kit (catalog ER211) was performed according to the manufacturer’s protocol to lyse virus and release DNA/RNA. RNA was extracted, then reverse-transcribed into cDNA, while DNA was extracted directly and analyzed by RT-qPCR. All primers used in the article were listed in Supplemental Table 3.

### Primary HBE ALI culture and virus infection

Primary HBEs at passage 3 or earlier were seeded at a density of 40,000 cells per well in Transwell inserts (Costar 6.5 mm Transwell, 0.4 μm Pore Polyester Membrane Inserts, catalog 3470) for expansion culture. The cells were cultured in expansion medium (PneumaCult-Ex Plus Medium, catalog 05040) until tight junctions were formed. Once tight junctions were established, the expansion medium in the upper chamber was discarded, and the medium in the lower chamber was replaced with ALI differentiation medium (PneumaCult-ALI Medium, catalog 05001). Cells were maintained under ALI conditions, with the medium being changed every 2 days. The differentiation process was carried out for 21 days, during which cell morphology and differentiation status were monitored. The fully differentiated cells were infected with HCoV-OC43, HCoV-229E, HCoV-NL63, or SARS-CoV-2 (MOI = 1), and fresh medium was changed 1 hour postinfection. Specific peptide was added into the medium every 24 hours. At 96 hours postinfection, cells in Transwell inserts were washed with 140 μL PBS per insert, and the wash fluid was collected to extract virus RNA followed by RT-qPCR. At the same time, cells in inserts were collected and fixed with 4% paraformaldehyde and permeabilized with 0.1% Triton X-100 and 3% BSA in PBS, and then immunofluorescence assay was performed to detect N protein. The fluorescence was measured using ImageJ (NIH).

### TEM

WT or *Hgs*-KO 17Cl-1 cells were grown on ACLAR films and infected with MHV. At 24 hpi the cells were rinsed with PBS and then fixed with 2.5% glutaraldehyde in PBS overnight. The fixed cells were rinsed with 0.1 M PB 2 times and then double-distilled water 2 times, each time for 6 minutes. They were submerged in an aqueous solution containing 1% osmium tetraoxide and 1.5% potassium ferricyanide for 1.5 hours and then rinsed with double-distilled water 3 times, each time for 6 minutes. The samples underwent a dehydration process in which the solution submerging the samples was sequentially replaced by 30%, 50%, 70%, 80%, 90%, 100%, 100%, and 100% ethanol solutions, with each solution submerging the sample for 6 minutes. The samples were then infiltrated sequentially with 100% acetone for 6 minutes 2 times, 3:1 acetone/EMbed 812 resin for 1 hour, 1:1 acetone/EMbed 812 resin for 2 hours, 1:3 acetone/EMbed 812 resin for 3 hours, and 100% EMbed 812 resin for 12 hours 2 times. The samples were embedded in EMbed 812 resin and underwent solidification at 45°C for 12 hours and then 60°C for 48 hours. Sectioning was performed with a Leica EM UC6 ultramicrotome. The sections were stained with 2% uranyl acetate for 15 minutes and 1% lead citrate for 5 minutes. Electron micrographs were acquired with an FEI Tecnai Spirit 120 kV transmission electron microscope operating at 100 kV accelerating voltage.

### Cytotoxicity assay

The cytotoxic effects of peptides and compounds on Huh7.5.1, 17Cl-1, Vero E6, and Calu-3 cells were measured by Cell Counting Kit-8 (CCK-8) assay. Briefly, a cell monolayer (1 × 10^4^) grown in 96-well plates was incubated with indicated concentrations of M146 or M161 peptides or compounds. After 24 hours, the culture medium was replaced with CCK-8 (Beyotime catalog C0039) solution in fresh medium at 37°C for 3 hours. The absorbance at 450 nm was measured by using a Spectrophotometer Multiskan SkyHigh with Touch Screen (Thermo Fisher Scientific) or BioTek Synergy H1 multimode reader (Agilent). The CC_50_ value of peptides or compounds to cells was calculated by GraphPad Prism 8.0.1 software. Critical commercial assays used in this study are provided in Supplemental Table 7.

### In vitro antiviral activity assay

Huh7.5.1, HRT-18, LLC-MK2, 17Cl-1, and Vero E6 cells were seeded in 96-well plates at densities of 4.5 × 10^4^, 4 × 10^4^, 2.5 × 10^4^, 2 × 10^4^, and 2 × 10^4^ cells/well, respectively, 1 day prior to infection. Cells were infected with HCoV-229E, HCoV-OC43, HCoV-NL63, MHV, WIV1, or SARS-CoV-2 at an MOI of 0.1. Serially diluted compounds were premixed with viruses, and 100 μL of each mixture was inoculated onto cell monolayers. At 24 or 48 hours postinoculation, IFA was performed using the Operetta CLS high-content analysis system. Inhibition rate = (1 – [infection rate of the test compound – cell control]/infection rate of the virus control) × 100%. Following the analysis of inhibition rates, the EC_50_ is determined using a 4-parameter fitting process. Compound inhibition and EC_50_ values were calculated from virus infection rates using GraphPad Prism 8.0.1 software. Data represent 1 of 3 independent experiments, each conducted with 8 concentration gradients in triplicate wells.

### SPR assay

SPR assay was performed on a Biacore 8K (Cytiva). M peptides were immobilized in 1 μM on a CM5 sensor chip to reach at least 700 resonance units, followed by flowing of hHGS 1-390-6 × HIS protein at varying concentrations in 1× PBS (pH 7.4). Binding studies were performed by passing 2-fold serial dilutions of purified hHGS 1-390-6 × HIS protein and measured by a multiple-cycle method with 120 s association and 600 s dissociation at a flow rate of 30 μL/min. For competitive inhibition experiments, the concentration of RTB is 200 μM. In addition, the direct interaction between hHGS 1-390-6 × HIS protein and RTB was detected using CM7. The resulting SPR sensorgrams were recorded and analyzed using the software provided by the vendors to extract the association and dissociation rate constants (*K_a_* and *K_d_*) and the binding affinity (*K_D_*). Each SPR experiment was repeated at least 3 times and analyzed by Prism 8.0.1 software.

### FP HGS-peptide interaction assays

FP assays were performed in black, 384-well microplates (Corning, 3575) with 20 μL of the assay solution per well, which contains 0.8 μM hHgs 1-390 protein and 180 nM FITC-peptide tracer. The optimal FP buffer was 50 mM HEPES, pH 7.2; 10 mM DTT; and 0.1 mM EDTA, pH 7.2, with additives (0.005% Tween-20). Ligand stocks were prepared through a serial dilution in DMSO and added to the wells. Assay mixture was incubated for 1 hour at room temperature before reading the plate. All data were collected on a microplate reader (Synergy H1). Excitation (485/20 nm) and emission (520/20 nm) filters were used for the FP measurements. All the experiments were independently repeated at least 3 times. The IC_50_ curves of compounds were analyzed in Prism 8.3.

### HTS of an in-house compound library

In the FP-based HTS assay, the compounds (10 mM in DMSO stock for a library of 5,000+ compounds) were transferred into each well of a 384-well plate that contains 0.8 μM HGS 1-390 protein and 180 nM FITC-peptide tracer using pintool at 33 μM final concentration. In each assay plate, the DMSO and unlabeled peptides were used as negative and positive controls, respectively. Free tracer wells (180 nM tracer only) were also set up as gain value in each assay plate. After incubation for 1 hour at RT, the FP was measured with an excitation at 485/20 nm and emission at 520/20 nm for FITC. The top 50 hit compounds were validated with FITC-peptide tracer in an FP assay, with each tested in 3 independent replicates.

### HDX

#### HDX-MS for peptide identification.

Peptides were identified using tandem MS (MS/MS) with a Q Exactive HF mass spectrometer (Thermo Fisher Scientific). Product ion spectra were acquired in data-dependent mode with the top 8 most abundant ions selected for the product ion analysis per scan event. The MS/MS data files were entered into pFind for high-confidence peptide identification.

#### HDX-MS analysis.

Ten microliters of hHGS (50 mM HEPES, pH 7.5; 50 mM NaCl) was incubated with and without the RTB at a 1:50 molar ratio (protein/ligand) for 0.5 hour before the HDX reacted at 4°C. Four microliters of protein/protein complex with ligand/peptide was diluted into 16 μL of D_2_O in exchange buffer (50 mM HEPES, pH 7.5; 50 mM NaCl) and incubated for various HDX time points (e.g., 0, 60, 300 s) at 4°C and quenched by mixing with 20 μL of ice-cold 3 M guanidine hydrochloride and 1% trifluoroacetic acid. Each quenched sample was immediately injected into the LEAP Pal 3.0 HDX platform. Upon injection, samples were passed through an immobilized pepsin column (2 mm × 2 cm) at 120 μL/min, and the digested peptides were captured on a C18 PepMap300 trap column (Thermo Fisher Scientific) and desalted. Peptides were separated with a 2.1 mm × 5 cm C18 separating column (1.9 μm Hypersil Gold, Thermo Fisher Scientific) with a linear gradient of 4%–40% CH_3_CN and 0.3% formic acid over 6 minutes. Sample handling, protein digestion, and peptide separation were conducted at 4°C. Mass spectrometric data were acquired using a Q Exactive HF mass spectrometer (Thermo Fisher Scientific) with a measured resolving power of 65,000 at *m/z* 400. HDX analyses were performed in triplicate for each preparation of a single protein-ligand complex. The intensity-weighted mean *m/z* centroid value of each peptide envelope was calculated and subsequently converted into a percentage of deuterium incorporation. Statistical significance for the differential HDX data is determined by a 2-tailed *t* test for each time point, a procedure that is integrated into the HDX Workbench software (50). Corrections for back exchange were made on the basis of an estimated 70% deuterium recovery and accounting for the known 80% deuterium content of the deuterium exchange buffer (51).

#### Data rendering.

The HDX data from all overlapping peptides were consolidated to individual amino acid values using a residue averaging approach. Briefly, for each residue, the deuterium incorporation values and peptide lengths from all overlapping peptides were assembled. A weighting function was applied in which shorter peptides were weighted more heavily and longer peptides were weighted less heavily. Each of the weighted deuterium incorporation values were then averaged to produce a single value for each amino acid. The initial 2 residues of each peptide, as well as proline residues, were omitted from the calculations.

### Statistics

The data were obtained from at least 3 independent experiments (*n* ≥ 3), and the representative data are presented as the mean ± SD as indicated and were corrected for multiple comparisons. One-way ANOVA and 2-tailed Student’s *t* tests were used to analyze differences in mean values. GraphPad Prism 8.0.1 software was used to calculate the *P* values, and significance is considered at *P* ≤ 0.05.

### Study approval

All animal studies were approved by the Guangzhou National Laboratory Animal Care and Use Committee and met stipulations of the NIH *Guide for the Care and Use of Laboratory Animals* (National Academies Press, 2011).

### Data availability

The data supporting the findings of this study are documented within the paper and are available in the Supporting Data Values file and from the corresponding authors.

Further information can be found in Supplemental Methods.

## Author contributions

XL performed the genome-wide CRISPRi screen, most viral experiments, and IF. RC performed the IB and co-IP and collaborated with KC and FL to construct the plasmids and KO cell lines. YC, YL, JZ, and JS carried out FP-based HTS and HDX analysis. RB performed the animal experiments. BT carried out the TEM experiments. YW and JZ helped with SARS-CoV-2 infection. PZ and LM helped with WIV1 infection. YT and QL helped with ALI-cultured HBEs. ZL initiated the study, directed the research, and wrote the manuscript. XL, RC, RB, BT, YC, KC, FL, YW, YT, QY, LM, FW, MZ, XQ, YL, JZ, PZ, XC, QL, XW, YS, YX, JZ, WJ, LH, JS, TX, and ZL discussed the data and reviewed the manuscript.

## Funding support

- Brain Science and Brain-like Intelligence Technology–National Science and Technology Major Project (2022ZD0211900 to YX).
- National Natural Science Foundation of China (92469107 to ZL; 82170473 to JS).
- Major Project of Guangzhou National Laboratory (GZNL2024A01011 to YX; GZNL2023A01008 to JS; SPRG22-002 to ZL, JS, and XC).
- National Key R&D Program of China (2024YFA1307400 to YX).
- Guangdong Province High-level Talent Youth Project (2021QN02Y939 to ZL; 2021QN020451 to JS).

## Supplementary Material

Supplemental data

Unedited blot and gel images

Supplemental table 1

Supporting data values

## Version 1 12/16/2025

In-Press Preview

## Version 2 03/02/2026

Electronic publication
