# Supplementary material for: Targeting the host factor HGS–viral membrane protein interaction in coronavirus infection
Source: J Clin Invest. 2025 Dec 16;136(5):e200225. doi: 10.1172/JCI200225 (PMC12948427; doi:10.1172/JCI200225)
Supplement: Supplemental data [file jci-136-200225-s269.pdf]

Supplementary materials for

Targeting the host factor HGS-viral membrane protein interaction in coronavirus infection

Xubing Long[1, 2, #], Rongrong Chen[3, 4, #], Rong Bai[2, 4, 7, #], Buyun Tian[1, 2, #], Yu Cao[2, #], Kangying Chen[2], Fuyu Li[2], Yiliang Wang[2], Yongjie Tang[2], Qi Yang[2], Liping Ma[2], Fan Wang[5], Maoge Zhou[2], Xianjie Qiu[1,2], Yongzhi Lu[1,2], Jie Zheng[6], Peng Zhou[2], Xinwen Chen[2], Qian Liu[2], Xuepeng Wei[2], Yongxia Shi[5, *], Yanhong Xue[3, *], Jincun Zhao[2, *], Wei Ji[3, *], Liqiao Hu[2, *], Jinsai Shang[1, 2, *], Tao Xu[2, 3, 4, *], Zonghong Li[1, 2, *]

[1]The First Affiliated Hospital of Guangzhou Medical University, School of Basic Medical Sciences, Guangzhou Medical University, Guangzhou, China

[2]Guangzhou National Laboratory, Guangzhou, China

[3]National Laboratory of Biomacromolecules, CAS Center for Excellence in Biomacromolecules, Institute of Biophysics, Chinese Academy of Sciences, Beijing, China

[4]College of Life Sciences, University of Chinese Academy of Sciences, Beijing, China

[5]State Key Laboratory of Respiratory Disease, Health Quarantine Institute of IQTC, Guangzhou Customs District, Guangzhou, China

[6]Shanghai Institute of Virology, Shanghai Jiao Tong University School of Medicine, Shanghai 200120, China

[7]Wuhan Institute of Virology, Chinese Academy of Sciences, Wuhan, China

# These authors contributed equally to this paper

*Correspondence: E-mail: 93240850@qq.com (Y. S.), xueyanhong@ibp.ac.cn (Y. X.), zhao_jincun@gzlab.ac.cn (J. Z.), jiwei@ibp.ac.cn (W.J.), hu_liqiao@gzlab.ac.cn (L. H.), shang_jinsai@gzlab.ac.cn (J. S.), xutao@ibp.ac.cn (T. X.) and li_zonghong@gzlab.ac.cn (Z. L.)

This file includes Materials and methods, Figure S1-8 and Table S2-7.

Materials and methods

Animals

Hgs$^{flox/-}$ mice (Strain NO. S-CKO-02885) and K18-hACE2-2A-CreERT2 mice (Strain NO. C001244) in the C57BL/6 background were purchased from Cyagen (Suzhou) Biological Information Technology Co, Ltd. These HGS-floxed mice were then crossed with K18-hACE2-2A-CreERT2 mice to produce double-transgenic offspring: K18-hACE2-2A-CreERT2/HGS-floxed mice. To achieve conditional *Hgs* KO specifically in hACE2-expressing cells, intraperitoneal tamoxifen injections were administered to induce Cre-mediated recombination. All mice were maintained under specific pathogen-free (SPF) conditions and were handled according to the guidelines of the institutional animal guidelines of the animal facilities. Mice were genotyped by PCR amplification of tail DNA using primers specific for Cre recombinase (5′-CATATTGGCAGAACGAAAACGC-3′; 5′-CCTGTTTCACTATCCAGGTTACGG-3′), HGS loxP1 sites (5′-AACCCTTAGAGGGAAACGCAAATC-3′; 5′-TCTAGGGTCCACAGCTAAACTCTC-3′) and HGS loxP2 sites (5′-CTGCTGCAAGGCTACAAGAGT-3′; 5′-ATCCTAATCCATGATCCCCTTTCT-3′).

A 15 mg/mL tamoxifen (Sigma-Aldrich, St. Louis, MO) solution was prepared by dissolving 30 mg tamoxifen in 2 mL corn oil (Sigma-Aldrich). Six-week-old K18-hACE2-2A-CreERT2/Hgs$^{flox/flox}$ mice were intraperitoneally injected with tamoxifen solution (75 mg/kg body weight) once daily for five consecutive days. Following induction for 10 days, mice were intranasally inoculated with 1×10^4 FFU SARS-CoV-2 Omicron BA.5 diluted in 50 µL DMEM. The mice were weighed and visually monitored once daily to score morbidity for 10 days. At 2 days post-infection (Dpi), a group of mice were euthanized and lung organ tissues were sampled for virological and histopathological analyses. Mice were euthanized when they became moribund or died because of difficulty eating or drinking. Randomization and allocation concealment were performed, and the operators of animal experiments were blinded to the group allocation. This work was conducted in the BSL-3 Laboratory of Guangzhou Customs District Technology Center.

The ROSA26-Cas9 mice (Strain NO. S23101012) were purchased from Cyagen (Suzhou) Biological Information Technology Co, Ltd. The C57BL/6JGpt mice were purchased from Zhuhai BesTest Biological Technology Co, Ltd. All animal experiments were approved by the Institutional Animal Care and Use Committee (IACUC) of Guangzhou National Laboratory (GZLab-AUCP-2023-01-A7), and performed in BSL-2 Lab of Guangzhou National Laboratory. The mice well fed in BSL-2 Laboratory for several days to adapt to the environment before performing experiments.

ROSA26-Cas9 mice (6-week-old) were randomly allocated and intravenously injected AAV-U6-mCherry-Control or AAV-U6-mCherry-sgHgs virus particles (1×10^{12} v.g./mL, 100 µL). After 2 weeks, the mice were inoculated intranasally with MHV (1.5

× 10^5 FFU). Viral challenge was operated under anesthesia to minimize animal suffering. After infection, the mice were weighed daily. At 5 dpi, mice were euthanized to excise the organs of liver and lung. The collected lung and liver tissues were subjected to examination of viral gRNA levels using RT-qPCR, virus titers by virus plaque assay, and assessment of pathological changes via HE staining.

Eight-week-old C57BL/6JGpt mice were randomly divided into four groups and intranasally inoculated with MHV virus (1.5 × 10^3 FFU). 2 h prior to MHV infection, each group received an intravenous injection of M-derived peptides at indicated concentration. Subsequently, mice were administered peptides via intravenous injection once daily for three consecutive days. Body weights were monitored daily. At 4 dpi, mice were euthanized, and lungs were collected for examination of viral RNA levels using RT-qPCR, virus titers by virus plaque assay, and assessment of pathological changes via HE staining. For tissue distribution analysis, at 1 h, 6 h, and 12 h post-tail vein injection of 300 μg FITC-peptide, nine organs were excised and imaged using a multimodal animal in vivo imaging system (AniView100, BLT).

Eight-week-old K18-hACE2-2A-CreERT2 C57BL/6JGpt mice were randomly assigned to groups and intranasally inoculated with either SARS-CoV-2 Omicron BA.5 virus (2 × 10^4 FFU) or HCoV-229E virus (6 × 10^4 FFU). Concurrently with inoculation, each group began receiving once-daily oral gavage of RTB or Molnupiravir at the indicated concentrations. Treatment continued for ten consecutive days for SARS-CoV-2 infection or two consecutive days for HCoV-229E infection. RTB (Selleck, S6441) was dissolved at 10, 20 or 30 mg/mL in 20% anhydrous ethanol and 80% corn oil. Molnupiravir (Selleck, S8969) was dissolved at 10 mg/mL in 5% DMSO and 95% PBS. Mice were monitored daily for weight changes and any clinical signs. A subset of mice was euthanized at 2 dpi for lung collection and subsequent virological and histopathological analyses. Additional mice were euthanized upon becoming moribund or if death occurred due to an inability to eat or drink. Infectious viral lung titers were quantified by fluorescent focus assay (FFA). All SARS-CoV-2 infection work was conducted in the BSL-3 laboratory of Guangzhou National Laboratory, while all HCoV-229E work was performed in the BSL-2 laboratory.

Eight-week-old C57BL/6JGpt mice were randomly divided into five groups and intranasally inoculated with MHV virus (6 × 10^4 FFU). 6 h post MHV infection, each group received once-daily oral gavage of RTB at the indicated concentrations. Treatment continued for four consecutive days. Mice were monitored daily for weight changes and any clinical signs, and euthanized at 4 dpi for lung collection and subsequent virological and histopathological analyses. All MHV work was performed in the BSL-2 laboratory.

A list of animal models used in this study is provided in Table S2.

Immunohistochemistry

The lung tissue induced by tamoxifen and the paraffin-coated sections of the non-induced lung tissues were dewaxed in xylene and hydrated in ethanol. The endogenous peroxidase was blocked using a 3% H_2O_2 solution. The antigen was retrieved by boiling in a citric acid solution (pH = 6.0), and non-specific binding sites were blocked with 3% BSA at room temperature for 30 min. The tissues were then incubated with anti-HGS antibody (1: 400, 10390-1-AP, Proteintech) at 4 °C overnight. After washing, the sections were conjugated with a horseradish peroxidase (HRP) antibody (1: 2000, ab205718, Abcam) at room temperature for 50 min. The tissues were developed using 3,3'-diaminobenzidine (DAB) reagent, counterstained with hematoxylin, dehydrated, and mounted.

Plasmids and peptides

Plasmids containing S protein of SARS-CoV-2 WT and SARS-CoV-1 were generously provided Dr. Shibo Jiang (Fudan University), S protein of SARS-CoV-2 BA4.6 and MERS-CoV were gifted by Dr. Shaobo Wang (Guangzhou National Laboratory). Pan-coronavirus VLP plasmids pCMV-MEN (HCoV-HKU1), pCMV-MEN (SARS-CoV-2), pCMV-MEN (MERS-CoV) and pCMV-MEN (HCoV-OC43) were provided from Prof. Guangxia Gao (Institute of Biophysics, Chinese Academy of Sciences). pcDNA3.1-N (SARS-CoV-2)-flag, pcDNA3.1-M (SARS-CoV-2) and pcDNA3.1-E (SARS-CoV-2)-HA were obtained from Dr. Binbin Ding (Guangzhou National Laboratory). pmCherry-N1-M (HCoV-HKU1), pmCherry-N1-M (SARS-CoV-1), pmCherry-N1-M (MERS-CoV) and pEGFP-N1-Hgs *(Greater horseshoe bat)* were synthesized by Rui Biotech, China. Pan-coronavirus structural protein cDNA fragments (S, M), mutations of S (B1.1.529), mutations of M (SARS-CoV-2) and Hgs of different species or mutations were amplified by Phanta MaxSuper-Fidelity DNA polymerase (Cat. No. P505-d2) and cloned into pLVX-mcherry, pLenti-GFP, pmCherry-N1, pEGFP-N1, pBFP-N1 or pcDNA3.1-flag vectors by ClonExpress II One Step Cloning Kit (Cat. No. C112-02). *Homo sapiens HGS* was cloned into the pET28a vector to protein expression and purification. Sequences of all the inserted DNAs were confirmed by sequencing (Rui Biotech, China). All sgRNAs were expressed in the pLG1 or plenti-CRISPR-V2 vector. The peptides (> 95% purity) were synthesized by DGpeptides Co., Ltd., Each peptide, containing 1 mg of powder, was stored at -80 °C. These peptides were dissolved in 1× PBS buffer to a final concentration of 1 mg/mL and diluted as needed. A list of peptides used in this study is provided in Table S4.

AAV and Lentivirus packaging and infection

HEK293T cells were co-transfected with pLVX or pLenti lentiviral expression plasmid or pLG1 sgRNA plasmid, and psPAX2 packaging plasmid, and pMD2.G envelope plasmid. The supernatants were harvested at 72 h post-transfection and concentrated by ultracentrifugation at 100,000 × *g* for 1 h, and then, the pellets were suspended in PBS to prepare 100-fold viral stocks. After preliminary titration, the lentiviral infections were performed following standard procedures.

The AAV2/9 was used to specifically knockdown *Hgs* in the liver of mice. In brief, sgRNA specific to the mouse *Hgs* or a scrambled control sequence was inserted into pAAV-CMV-mCherry and confirmed via sequencing. The sgRNA sequences used for targeting mouse *Hgs* and the scrambled control were GTACTTGGGTTCGTTCCGGA and ACGGAGGCTAAGCGTCGCAA, respectively. Recombinant AAV was produced by a triple-plasmid transfection system comprising pAAV-PC2 Vector, pHelper Vector and pAAV-CMV-sgRNA-mCherry. The viruses were purified using gradient ultracentrifugation and the virus titers were determined by RT-qPCR.

Generation of KO or knockdown cell lines

The KO cell lines were generated via CRISPR-Cas9 or CRISPRi system. sgRNAs targeting the indicated genes were designed by the online tool (https://chopchop.cbu.uib.no/). The corresponding DNA was synthesized and cloned into lenti-CRISPR-v2 puro, lenti-CRISPR-v2 BSD vector, or pLG-1 vector. 17Cl-1 and Huh7.5.1 cells were infected with lentivirus collected from HEK293T cells and incubated for 48 h with 8 μg/mL polybrene. Transduced cells were selected with 2 μg/mL puromycin and 30 μg/mL blasticidin (Cat. No. ST551, Cat. No. ST018) for 2 weeks. Single cell colonies were verified by IB analysis and sequencing of the PCR products. The sgRNA sequences were provided in Table S5.

Co-IP and IB

For endogenous co-IP, Vero E6 cells (2×10^6 cells) were seeded into a 10 cm dish and infected with SARS-CoV-2 (MOI = 0.5). After 24 h, the total cells were harvested and lysed in 1mL ice-cold IP-lysis buffer (50 mM Tris-HCl pH 7.4, 5 mM EDTA, 40 mM β-sodium glycerophosphate, 30 mM NaF, 1 mM PMSF, 1 mM Na_3VO_4, 10% glycerol, 1.0% NP-40, and 150 mM NaCl) in the presence of a protease inhibitor cocktail (Roche) and phosphatase inhibitors. For Co-IP, one 10 cm dish of HEK293T cells was transfected with 10-15 ug plasmid for 48 h, and then, the cells were collected and lysed in 1mL ice-cold NP-40 lysis buffer. Cell lysates were precleaned and incubated with anti-HGS, anti-FLAG or anti-HA antibody and protein A/G agarose beads at 4 °C overnight. After washing five times, the immunoprecipitated complexes were sampled with SDS loading buffer and subjected to IB.

Alternatively, the Pan-coronavirus VLPs in the supernatants were precipitated with a final concentration of 10% (m/v) TCA at 4 °C overnight. After washing two times with acetone and one time with methanol, the protein pellets were dissolved in SDS loading buffer and subjected to IB.

For IB, the samples were separated by SDS-PAGE and transferred to NC membranes. The membranes were blocked in 5% non-fat milk in PBST (1 × PBS with 0.05% tween 20) buffer at room temperature for 1 h and then incubated with primary antibodies at 4 °C overnight, and subsequently incubated with the secondary antibodies (IRDye 680/800, LI-COR Biosciences) at room temperature in the dark for 2 h. The images

were visualized and captured by the Odyssey M system (LI-COR Biosciences). Relative protein levels were measured by ImageJ and analyzed by the GraphPad Prism (8.0.1). A list of antibodies used in this study is provided in Table S6.

Protein purification and *in vitro* binding assay

For recombinant HGS protein, the cDNA of human *HGS* was cloned into pET28a vector with C-terminal 6 × HIS-tag and expressed in *Escherichia coli* BL21 (DE3) with 0.4 mM IPTG at 16 °C for 16 h. Bacterium were collected and lysed by sonication in PBS buffer containing 400 mM NaCl, 20 mM imidazole and protease inhibitors on ice. Supernatants were collected after centrifugation at 12,000 × *g* for 30 min at 4 °C, then was incubated with Ni-NTA agarose beads (Smart-lifesciences) for 2 h at 4 °C. The HIS-tagged HGS was eluted by elution buffer (PBS containing 400 mM NaCl and 250 mM imidazole). The following proteins: GST-FLAG (Cat. No. Ag2329), GST-Spike RBD (Cat. No. Ag30689), GST-M (101-222aa) (Cat. No. Ag30691) were purchased from Proteintech Group, lnc., and M-FLAG protein was gifted from Dr. Xuepeng Wei (Guangzhou National Laboratory).

For in vitro binding assay, anti-FLAG nanobody agarose beads were co-incubated with GST-FLAG or M-FLAG protein and Hgs-6 × HIS protein; Glutathione Sepharose (Cat. No. 17075605) was co-incubated with GST-FLAG or GST-M (101-222 aa) protein and HGS-6 × HIS protein. The beads were overnight at 4 °C, and washed three times with lysis buffer (1 × PBS buffer, contain 400 mM NaCl, 2.5 mM EDTA, 0.2% triton) to remove nonspecifically binding protein. The samples were then subjected to immunoblotting. A list of proteins used in this study is provided in Table S4.

RT-qPCR

The extraction of total RNAs was performed using TRIzol reagent (Invitrogen). Subsequently, 1 μg of total RNA per sample was reverse transcribed into cDNA using HiScript II Q Select RT SuperMix for qPCR. RT-qPCR was performed using the TB Green® Premix Ex Taq™ II (Tli RNaseH Plus) (Cat. No. RR820A) and calculated by using the $2^{-\Delta\Delta Ct}$ method with GAPDH as an internal reference gene. The qPCR was conducted with a CFX96 Touch System (CFX96 Touch Real-Time PCR Detection System, Bio-Rad Laboratories (Shanghai) Co., Ltd.). The primer sequences for quantitative measurement were shown in Table S3.

IF

Cells infected with virus were fixed with 4% paraformaldehyde at room temperature for 15 min and permeabilized with 0.1% Triton X-100 and 3% BSA in PBS at room temperature for 1 h. Cells were then incubated with specific antibodies at 4 °C overnight, followed by an Alexa Fluor-labeled secondary antibody at room temperature in the dark for 2 h. Images of the cells were taken using a NIKON A1 inverted microscope or Carl

Zeiss LSM 980. Manders' Coefficient was calculated using ImageJ with the Coloc 2 plug-in to quantify the fraction of one protein that overlapped with another.

Virus Plaque Assay

For intracellular and extracellular mature MHV titer determination, 100 μL medium with mature virus was collected and then diluted with medium and added into cell culture to infect 2×10^5 17Cl-1 cells at 37 °C for 2 h. 4 mL of overlay solution was added to each well. After 72 h, the overlay solution was discarded, and crystal violet solution was added. The plates were gently shaken for 2 h, then washed with water to remove the crystal violet stain. The total FFU was determined and represented.

To determine the MHV titer in liver and lung tissue homogenates, the samples were diluted 4-fold with PBS, followed by repeated freezing and thawing in liquid nitrogen. The samples were centrifuged at 10,000 × *g* for 10 min at 4 °C and the supernatant was collected. Next, 100 μL of the supernatant was added to L2 cells in a 12-well plate, with the cells at approximately 90% confluence (L2 cells were kindly provided by Prof. Deyin Guo, Guangzhou National Laboratory), and incubated at 37 °C for 2 h. 4 mL of overlay solution was added to each well. After 72 h, the overlay solution was discarded, and crystal violet solution was added. The plates were gently shaken for 2 h, then washed with water to remove the crystal violet stain. The total FFU of lung and liver tissue was determined and represented.

Focus forming assay (FFA)

Vero E6 cells were seeded 2×10^4 cells/well in 96-well plates one day before infection. Lung homogenate was serially diluted and used to inoculate Vero E6 cells at 37 °C for 1 h. Inocula were then removed before adding 125 μL per well 1.6% carboxymethylcellulose warmed to 37 °C. After 24 h, cells were fixed with 4% paraformaldehyde and permeabilized with 0.2% Triton X-100. Cells were then incubated with a rabbit anti-SARS-CoV-2 N protein polyclonal antibody (Cat. No.: 40143-T62, Sino Biological, Inc. Beijing), followed by an HRP-labeled goat anti-rabbit secondary antibody (Cat. No. 109-035-088, Jackson ImmunoResearch Laboratories, Inc. West Grove, PA). The foci were visualized by TrueBlue Peroxidase Substrate (cat. no. 50-78-02, KPL, Gaithersburg, MD), and counted with an ELISPOT reader (Cellular Technology Ltd. Cleveland, OH). Viral titers were calculated as FFU per mL.

Immunological Fluorescence Assay (IFA)

Huh7.5.1, HRT-18, LLC-MK2, 17Cl-1 and Vero E6 cells were seeded 4.5×10^4, 4×10^4, 2.5×10^4, 2×10^4 and 2×10^4 cells/well in 96-well plates one day before infection, separately. Cells were infected with HCoV-229E, HCoV-OC43, HCoV-NL63, MHV, SARS-CoV-2 or WIV1 at MOI = 0.1, respectively. After 24 h or 48 h, cells were fixed with 4% paraformaldehyde and permeabilized with 0.2% Triton X-100. Cells were then incubated with a rabbit anti-229E-N, OC43-N, NL63-N, a human anti-MHV-N or a

rabbit anti-SARS-CoV-2 N protein polyclonal antibody, followed by an Alexa Fluor 488-labeled goat anti-rabbit or human secondary antibody. Images of the cells were taken and infection of virus was calculated with the Operetta CLS™ high-content analysis system.

BFA treatment

70–80% confluent 17Cl-1 cells were treated with 5 μg/mL final BFA (Catalog no #HY-16592; MCE) for 40 min at 37°C. BFA was washed out by three brief rinses in PBS and cells were then incubated at 37°C for 5 h before fixation with 4% PFA.

Supplementary figure and figure legends

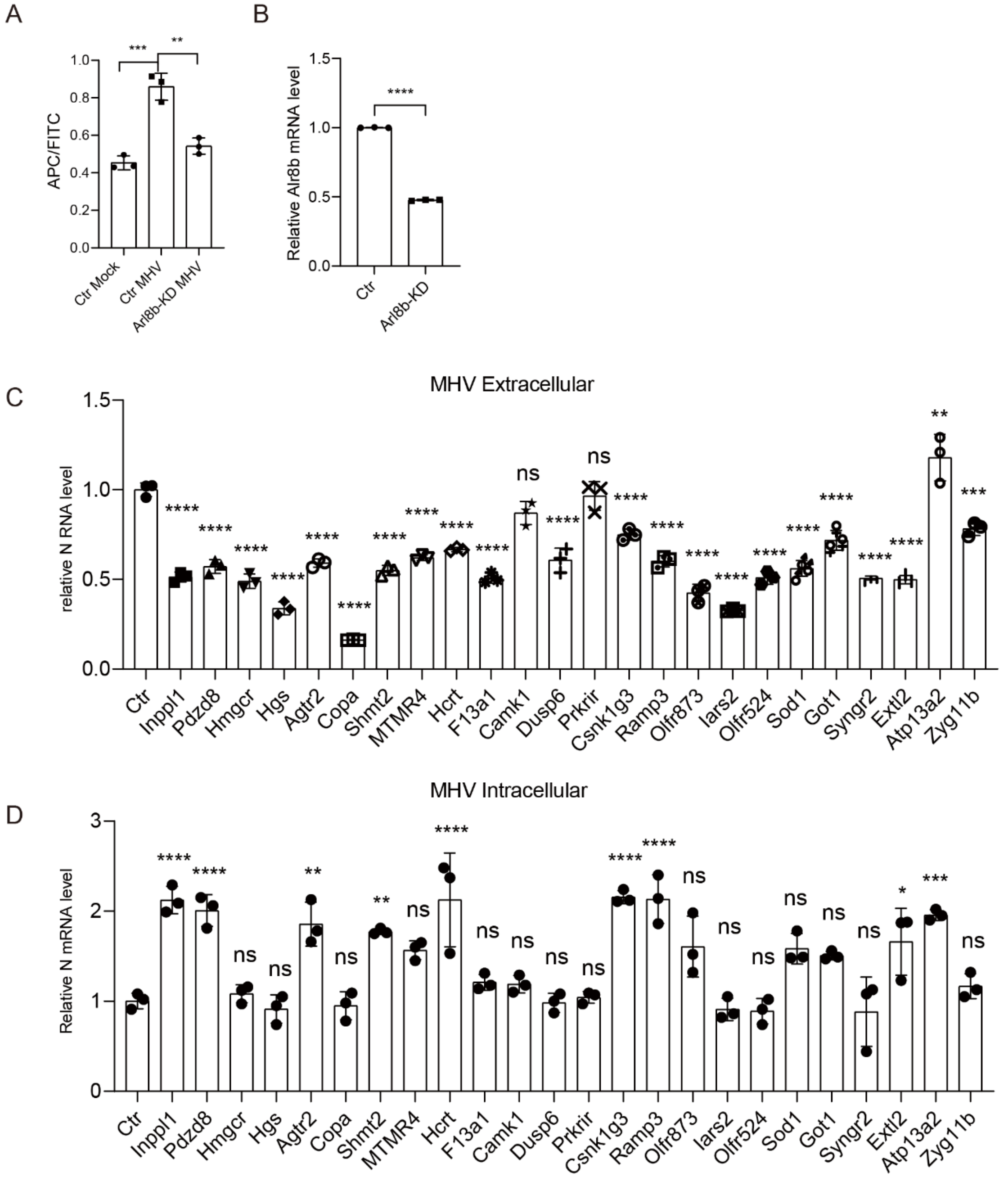

Figure S1. Genome-wide CRISPRi screens identify host factors for pan-coronavirus assembly and egress.

(A-B) Flow cytometry analysis of cell surface and total LAMP1 in Ctr and *Arl8b* knockdown 17Cl-1 cells infected with or without MHV at MOI = 1 for 7 h (A). RT-qPCR analysis of *Arl8b* expression in the Ctr and *Arl8b* knockdown 17Cl-1 cells (B). N = 3 independent biological replications.

(C-D) Validation of high-confidence host factors. RT-qPCR analysis of extracellular (C) and intracellular (D) viral gRNA levels in dCas9-KRAB expressing 17Cl-1 cells transduced with specific targeted sgRNA after infected with MHV (MOI = 1) for 16 h. N = 3 independent biological replications.

Data are the mean ± SD. Significance testing for (A, C and D) was performed with 1-way ANOVA and Tukey's multiple comparison test. Significance testing for (B) was performed with a 2-tailed t test. $^{*}P \leq 0.05$, $^{**}P \leq 0.005$, $^{***}P \leq 0.0005$, $^{****}P \leq 0.0001$, ns, no significance.

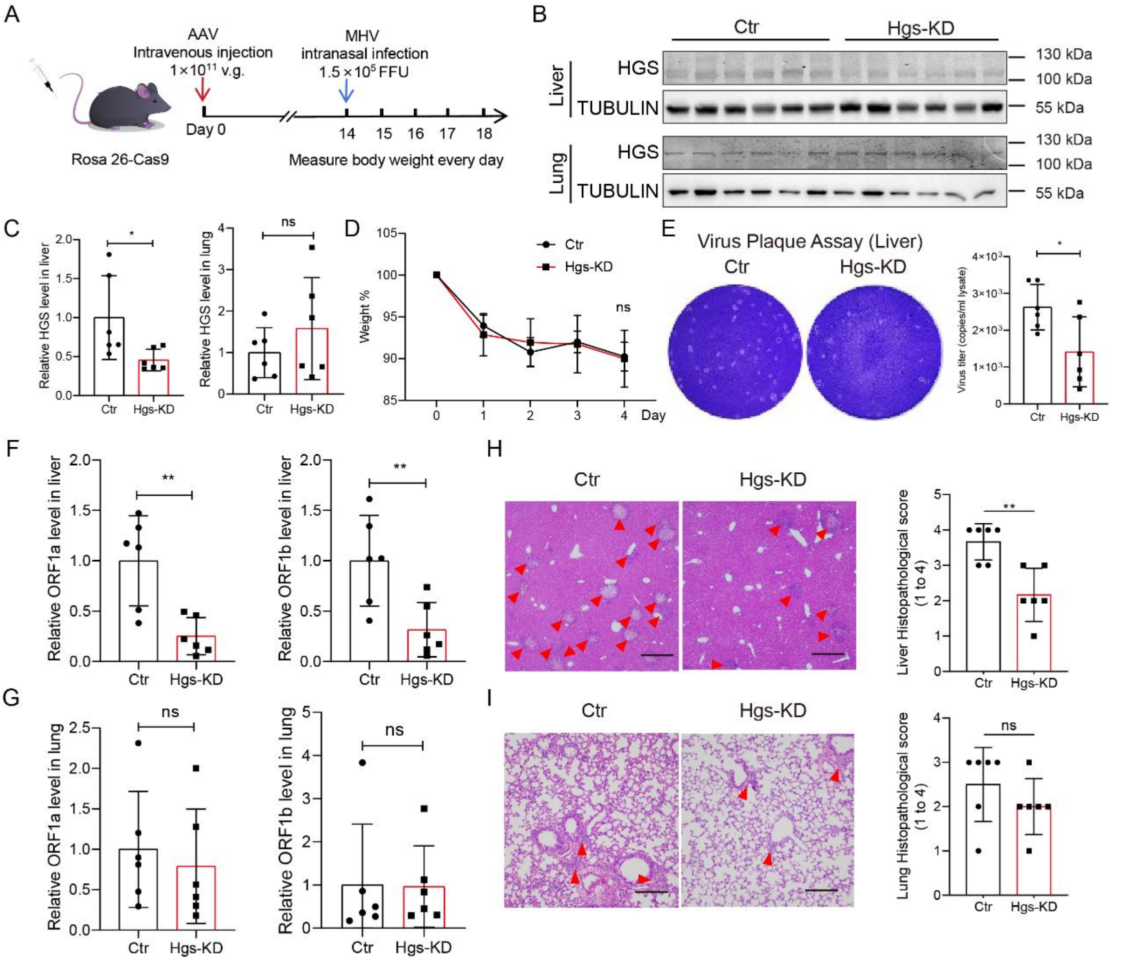

Figure S2. HGS facilitates MHV infection *in vivo*.

(A) Schematic illustrating MHV infection in the liver-specific *Hgs* knockdown mice. AAV (1×10^{11} v.g.) with scrambled sgRNA or *Hgs* specific targeted sgRNA intravenous injection of Rose26-Cas9 mice at Day 0, and MHV intranasal infection (1.5×10^5 FFU) at Day 14. Measure body weight for 5 days upon infection. N = 6.

(B-C) IB analysis of liver and lung HGS expression in the Ctr and *Hgs* knockdown mice (B). Tubulin was used as an internal reference protein. Quantitative analysis of relative HGS protein level in liver and lung (C). N = 6.

(D) Body weight loss in the Ctr and *Hgs* knockdown mice post MHV infection for 5 days. N = 6.

(E) Viral titration by plaque assay with the supernatant of homogenized liver tissues of the Ctr and *Hgs* knockdown mice on day 4. N = 6.

(F-G) RT-qPCR analysis of liver (F) and lung (G) MHV viral ORF1a and ORF1b levels in the Ctr and *Hgs* knockdown mice on day 4. *Gapdh* was used as an internal reference gene. N = 6.

(H-I) HE staining analysis of liver (H) and lung (I) tissue in the Ctr and liver-specific *Hgs* knockdown mice. Quantitative analysis of pathological severity scores based on the number of affected area in liver and lung tissues. N = 6. Scale bar = 200 μm

Data are the mean ± SD. Significance testing was performed with a 2-tailed t test. $*P \leq 0.05$, $**P \leq 0.005$, $***P \leq 0.0005$, $****P \leq 0.0001$, ns, no significance.

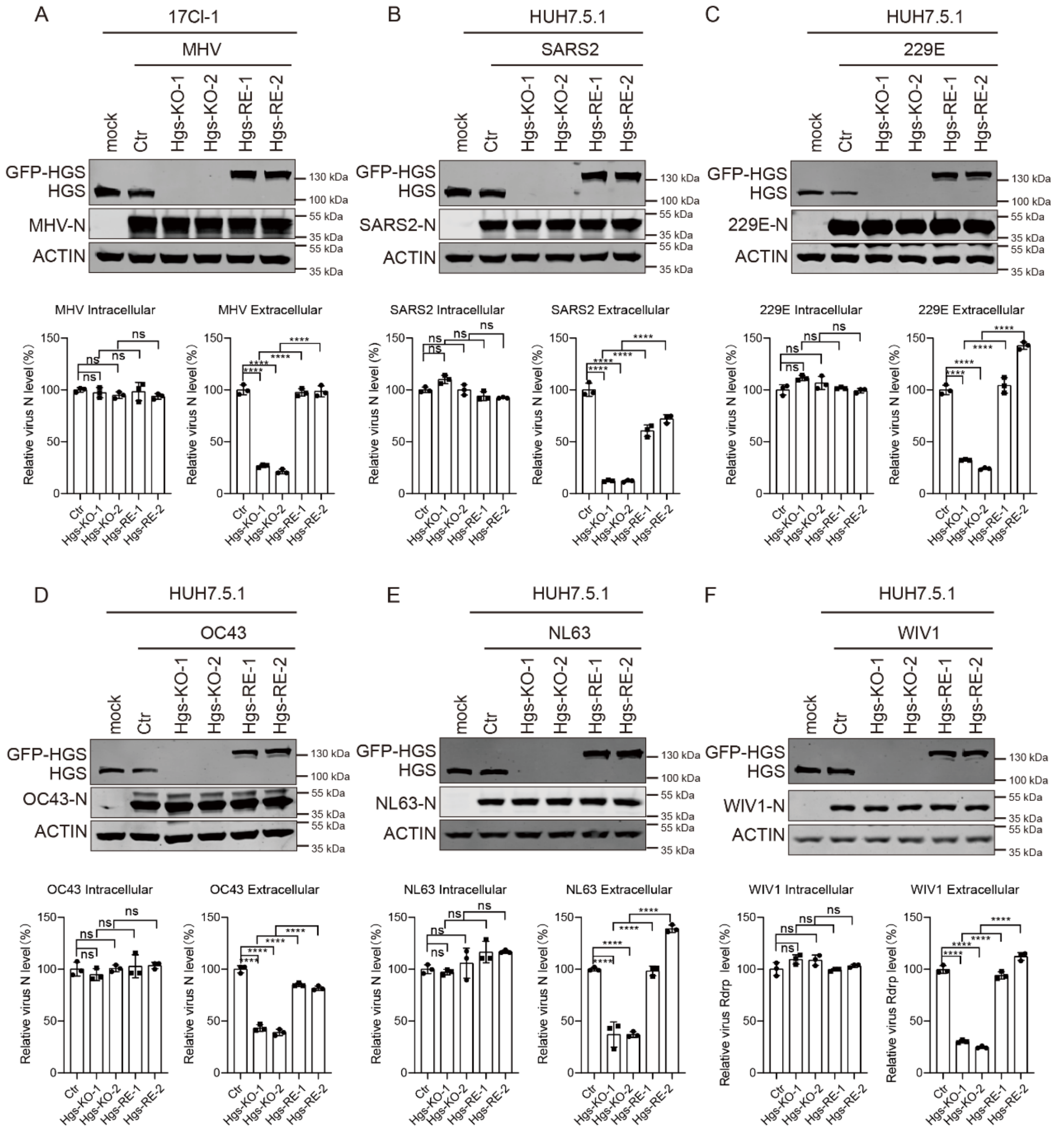

Figure S3. HGS regulates pan-coronavirus assembly and egress.

(A-F) RT-qPCR analysis of intracellular (lower left panel) and extracellular (lower right panel) viral gRNA levels in the Ctr, two *Hgs*-KO clones and their respective *Hgs*-recused cells after infected with MHV (17Cl-1 cells for 16 h, Multiplicity of infection, MOI = 1, A), SARS-CoV-2 (Huh7.5.1 cells for 24 h, MOI = 1, B), HCoV-229E (Huh7.5.1 cells for 24 h, MOI = 1, C), HCoV-OC43 (Huh7.5.1 cells for 24 h, MOI = 1, D), HCoV-NL63 (Huh7.5.1 cells for 24 h, MOI = 1, E) and WIV1 (Huh7.5.1 cells for 24 h, MOI = 1, F). IB analysis of HGS and N proteins expression in the Ctr, two *Hgs*-KO clones and their respective *Hgs*-rescued cells (upper panel). Actin was used as an internal reference protein. Molecular weights are in kDa. N = 3 independent biological replications.

Data are the mean ± SD. Significance testing for (A-F) was performed with 1-way ANOVA and Tukey's multiple comparison test. *P ≤ 0.05, **P ≤ 0.005, ***P ≤ 0.0005, ****P ≤ 0.0001, ns, no significance.

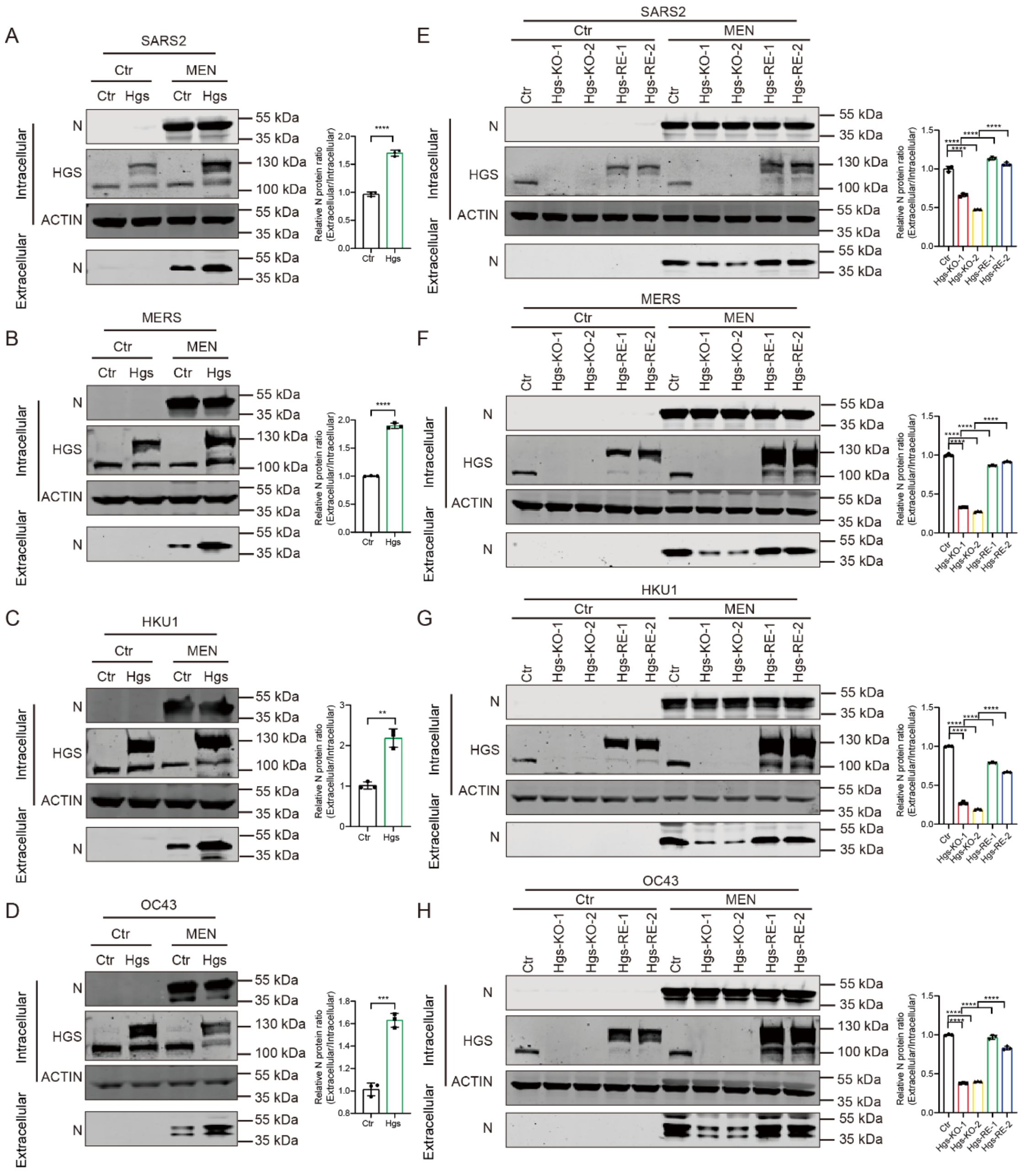

Figure S4. HGS facilitates VLP production.

Representative IB analysis of N protein for indicating SARS-CoV-2 (A, E), MERS (B, F), HCoV-HKU1 (C, G) and HCoV-OC43 (D, H) VLP production in the *HGS* overexpression (A-D), two *Hgs*-KO clones and their respective *HGS*-recused (E-H) cells. HEK293T cells were transfection with VLP system with HGS-GFP or sgRNA targeted *HGS* for 48 h. The extracellular secreted protein and intracellular cell lysis were examined by IB with the indicated antibody. Molecular weights are in kDa. N = 3 independent biological replications.

Data are the mean ± SD. Significance testing for (E-H) was performed with 1-way ANOVA and Tukey's multiple comparison test. Significance testing for (A-D) was performed with a 2-tailed t test. $^*P \leq 0.05$, $^{**}P \leq 0.005$, $^{***}P \leq 0.0005$, $^{****}P \leq 0.0001$, ns, no significance.

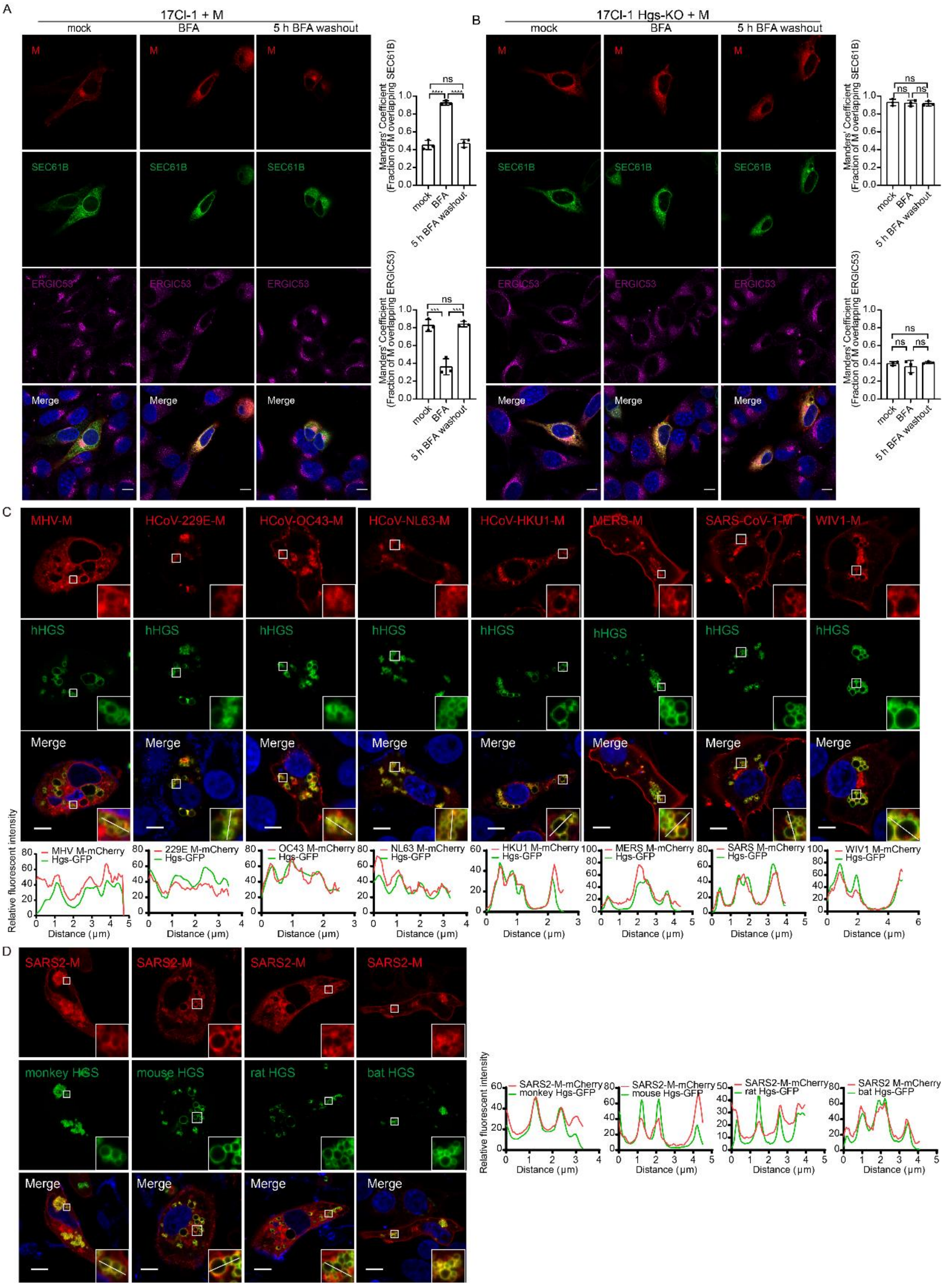

Figure S5. HGS promotes the flow of M protein out of ER and co-localizes with pan-coronavirus M proteins.

(A-B) Representative IF analysis of the colocalization of SARS-CoV-2 M with SEC61B or ERGIC53 in WT (A) and *Hgs*-KO (B) 17Cl-1 cells under mock, BFA treatment (5 μg/mL) or BFA washout for 5 h after treatment. Scale bar, 10 μm. Quantitative image analysis of M-SEC61B or M-ERGIC53 colocalization using

Manders' Coefficient. N = 3 independent biological replications.

(C) Representative IF analysis of the co-localization between *Homo sapiens (Human)* HGS and various coronavirus M proteins, including MHV, HCoV-229E, HCoV-OC43, HCoV-NL63, HCoV-HKU1, MERS, SARS-CoV-1, and WIV1. Scale bar, 10 μm. N = 3 independent biological replications.

(D) Representative IF analysis of the co-localization between SARS-CoV-2 M protein and different species HGS, including *Chlorocebus sabaeus (Green monkey) (Cercopithecus sabaeus)*, *Rattus norvegicus (Rat)*, *Mus musculus (Mouse)* and *Rhinolophus ferrumequinum (Greater horseshoe bat)*. Scale bar, 10 μm. N = 3 independent biological replications.

Data are the mean ± SD. Significance testing for (A and B) was performed with 1-way ANOVA and Tukey's multiple comparison test. *P ≤ 0.05, **P ≤ 0.005, ***P ≤ 0.0005, ****P ≤ 0.0001, ns, no significance.

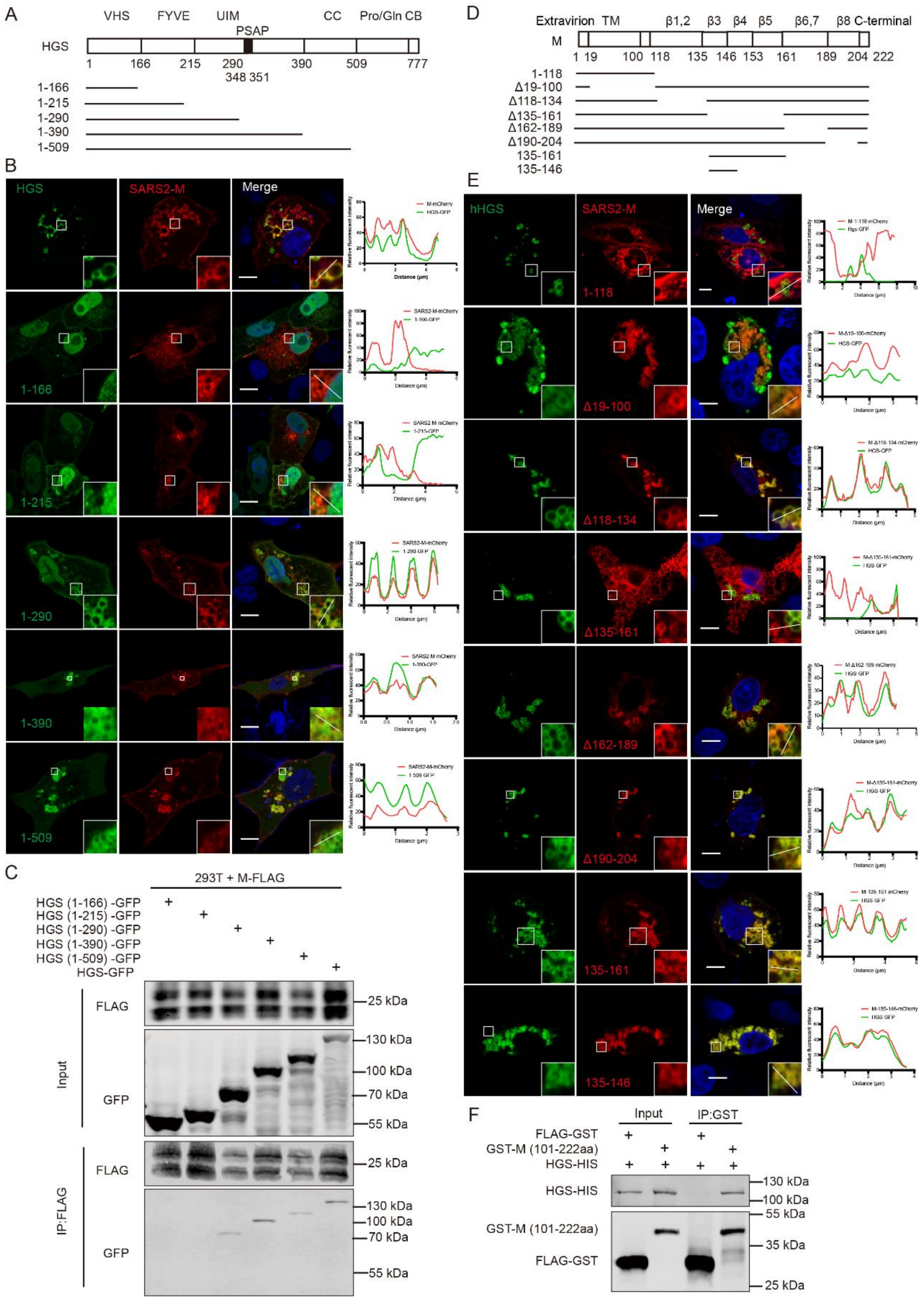

Figure S6. The N-terminal of HGS interacts with the intravirion domain of SARS-CoV-2 M protein.

(A) Schematic illustrating HGS truncations.

(B) Representative IF analysis of the co-localization between M and various truncations of HGS, including HGS (1-166), HGS (1-215), HGS (1-290), HGS (1-390) and HGS

(1-509). Scale bar, 10 µm. N = 3 independent experiments.

(C) Co-IP analysis of the interaction of M with various truncations of HGS. Proteins were transiently expressed in HEK293T cells, and immunoprecipitates pulled down by FLAG antibody were analyzed by IB with the indicated antibodies. Input represents 5% of the total cell extract used for immunoprecipitation. Molecular weights are in kDa. N = 3 independent biological replications.

(D) Schematic illustrating SARS-CoV-2 M truncations.

(E) Representative IF analysis of the co-localization between HGS and various truncations of M, including M (1-118), M (Δ19-100), M (Δ118-134), M (Δ135-161), M (Δ162-189), M (Δ190-204), M (135-161) and M (135-146). Scale bar, 10 µm. N = 3 independent biological replications.

(F) *In vitro* pull-down analysis of the interaction between HGS and the intravirion domain of M. Purified HGS and the intravirion domain of M proteins were subjected to pull-down assay, and pull-down samples by FLAG antibody were analyzed by IB with the indicated antibodies. FLAG-GST protein was used as a negative control. Input represents 5% of the total proteins used for pull-down. Molecular weights are in kDa. N = 3 independent biological replications.

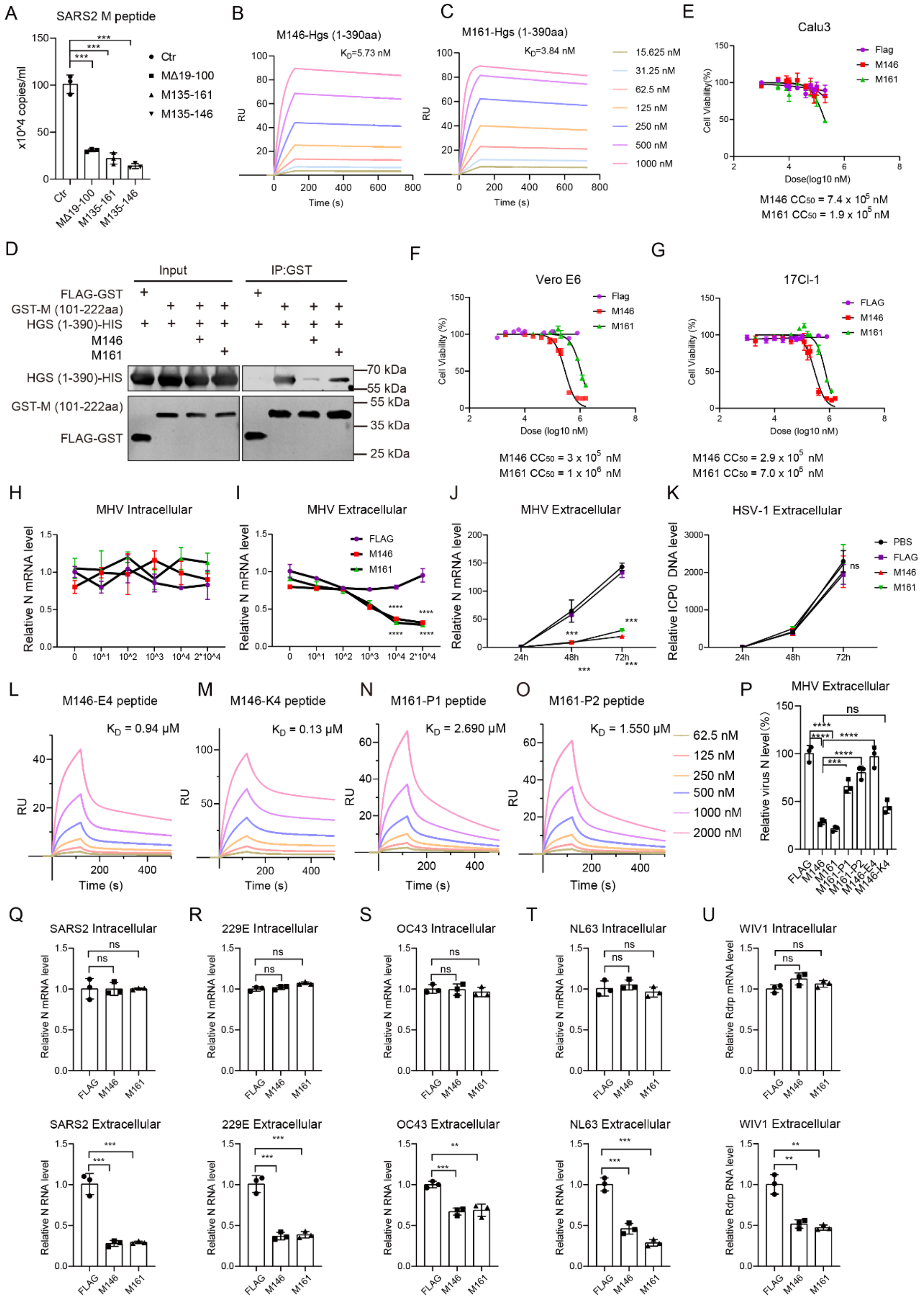

Figure S7. M-derived peptides specifically bind HGS and inhibit pan-coronavirus egress *in vitro*.

(A) Plaque assay analysis of extracellular MHV titer levels in the overexpression of SARS-CoV-2 M truncations 17Cl-1 cells. N=3 independent biological replications.

(B-C) SPR analysis of the binding affinity between HGS and peptides M146 (B) or

M161 (C). N = 3 independent biological replications.

(D) *In vitro* pull-down analysis of the competition binding of HGS between peptides M146 and M161 with the intravirion domain of M. Purified HGS (1-390) and the intravirion domain of M proteins with or without peptides M146 or M161 were subjected to pull-down assay, and pull-down samples by GST antibody were analysed by IB with the indicated antibodies. FLAG-GST protein was used as a negative control. Input represents 5% of the total proteins used for pull-down. Molecular weights are in kDa. N = 3 independent biological replications.

(E-G) CCK-8 analysis of the cytotoxic effects of M146 and M161 peptides on Vero E6, 17Cl-1 and Calu-3 Cells. N = 3 independent biological replications.

(H-I) RT-qPCR analysis of intracellular (H) and extracellular (I) viral gRNA levels in the different doses of FLAG, M146 or M161 peptides treated 17Cl-1 cells after infected with MHV (MOI = 1) for 24 h. N = 3 independent biological replications.

(J-K) RT-qPCR analysis of extracellular MHV viral gRNA or HSV-1 viral gDNA levels in the FLAG, M146 or M161 peptides treated (10^4 nM) 17Cl-1 cells after infected with MHV (MOI = 1) for 24 h, 48 h or 72 h. N = 3 independent biological replications.

(L-O) SPR analysis of the binding affinity between HGS and mutated peptides M146-E4 (L), M146-K4 (M), M161-P1 (N) and M161-P2 (O). N = 3 independent biological replications.

(P) RT-qPCR analysis of extracellular MHV viral gRNA in the FLAG, M146, M161, M146-E4, M146-K4, M161-P1 and M161-P2 treated (10^4 nM) 17Cl-1 cells after infected with MHV (MOI = 1) for 24 h. N = 3 independent biological replications.

(Q-U) RT-qPCR analysis of intracellular and extracellular SARS-CoV-2 (MOI = 0.5) (Q), HCoV-229E (MOI = 1) (R), HCoV-OC43 (MOI = 1) (S), HCoV-NL63 (MOI = 1) (T) and WIV1 (MOI = 1) (U) viral gRNA levels in infected Vero E6 cells treated with 10^4 nM FLAG, M146 or M161 peptides for 24 h. N = 3 independent biological replications.

Data are the mean ± SD. Significance testing was performed with 1-way ANOVA and Tukey's multiple comparison test. ***$P \leq 0.0005$, ****$P \leq 0.0001$, ns, no significance.

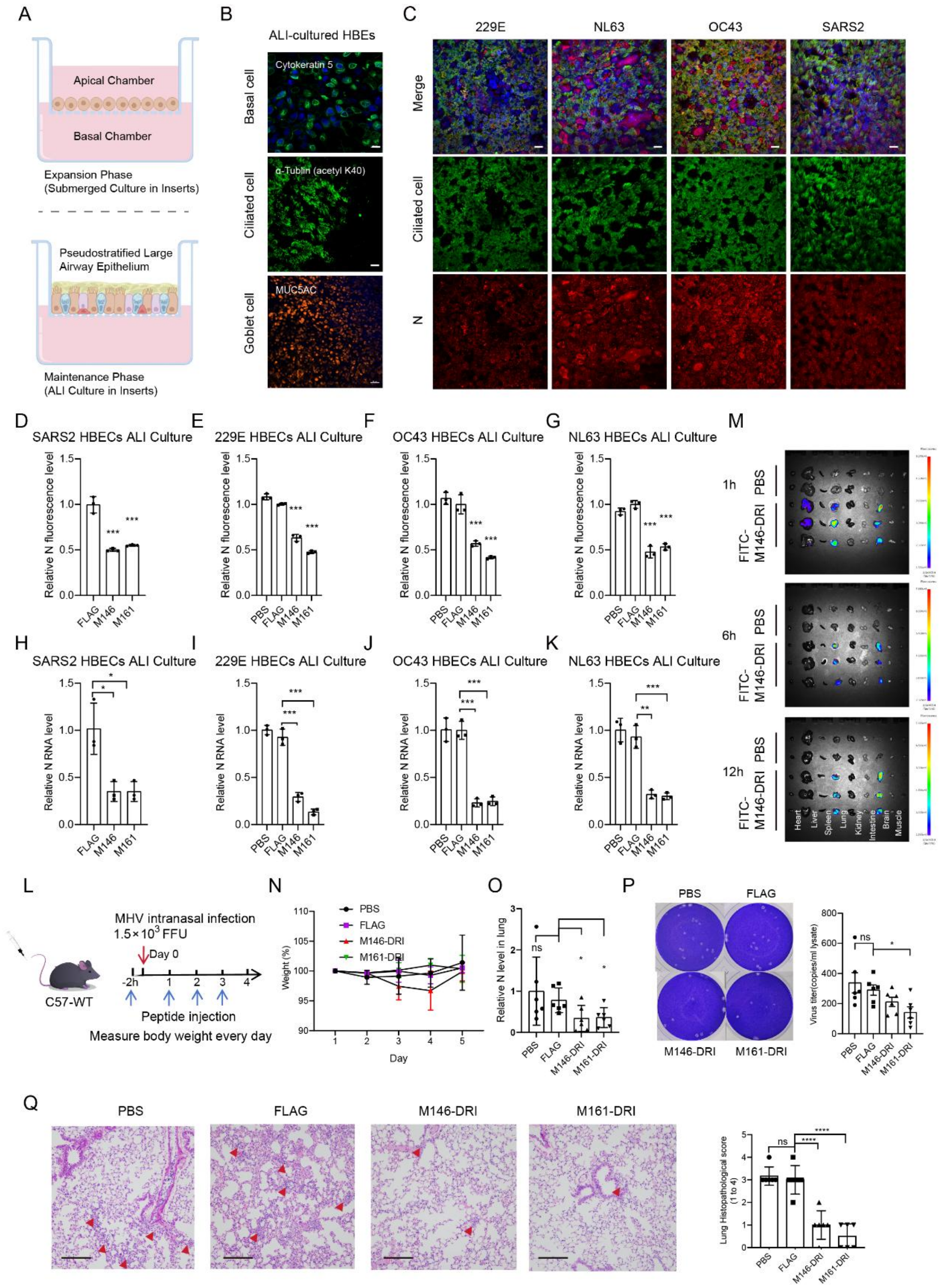

Figure S8. M-derived peptides alleviate the coronavirus infection in ALI-cultured HBEs and *in vivo*.

(A) Schematic illustrating primary HBEs in ALI differentiation.

(B) Representative IF analysis of basal cell, ciliated cell and goblet cell in the fully differentiated ALI-cultured HBEs. N = 3 independent biological replications.

(C) Representative IF analysis of HCoV-229E (MOI = 1, hpi = 96 h), HCoV-NL63 (MOI = 1, hpi = 96 h), HCoV-OC43 (MOI = 1, hpi = 96 h), SARS-CoV-2 (MOI = 0.5, hpi = 96 h) infected ALI-cultured HBEs. N = 3 independent biological replications.

(D-G) Quantitative analysis of relative N protein fluorescence levels in the SARS-CoV-2 (MOI = 0.5) (D), HCoV-229E (MOI = 1) (E), HCoV-OC43 (MOI = 1) (F) and HCoV-NL63 (MOI = 1) (G) infected ALI-cultured HBEs treated with 10^4 nM FLAG, M146 or M161 peptides for 96 h. N = 3 independent biological replications.

(H-K) RT-qPCR analysis of extracellular viral gRNA levels in the SARS-CoV-2 (MOI = 0.5) (H), HCoV-229E (MOI = 1) (I), HCoV-OC43 (MOI = 1) (J) and HCoV-NL63 (MOI = 1) (K) infected ALI-cultured HBEs treatment with 10^4 nM FLAG, M146 or M161 peptides for 96 h. N = 3 independent biological replications.

(L) Schematic illustrating MHV-infected mice treatment with HGS-targeted peptides. MHV-infected mice were pre-treated with PBS, FLAG, M161-DRI and M146-DRI peptides (15 mg/kg body weight) for 2 h, afterwards every 24 h for 4 days.

(M) *Ex vivo* fluorescence imaging analysis of the distribution of the FITC-labeled M146 peptide in different organs after intravenous injection for 1 h, 6 h, and 12 h, including the heart, liver, spleen, lung, kidney, intestine, brain and muscle.

(N) Measure body weight for 5 days. N = 6.

(O) RT-qPCR analysis of MHV viral N levels in the lung of the mice treatment with PBS, FLAG, M146-DRI or M161-DRI. *Gapdh* was used as an internal reference gene. N = 6.

(P) Viral titration by plaque assay with the supernatant of homogenized lung tissues of the mice treatment with PBS, FLAG, M146-DRI or M161-DRI on day 4. N = 6.

(Q) HE staining analysis of lung tissue in the PBS, FLAG, M146-DRI or M161-DRI treated mice. Quantitative analysis of pathological severity scores based on the percentage of affected area in lung tissues. N = 6.

Data are the mean ± SD. Significance testing was performed with 1-way ANOVA and Tukey's multiple comparison test. $*P \leq 0.05$, $**P \leq 0.005$, $***P \leq 0.0005$, $****P \leq 0.0001$, ns, no significance.

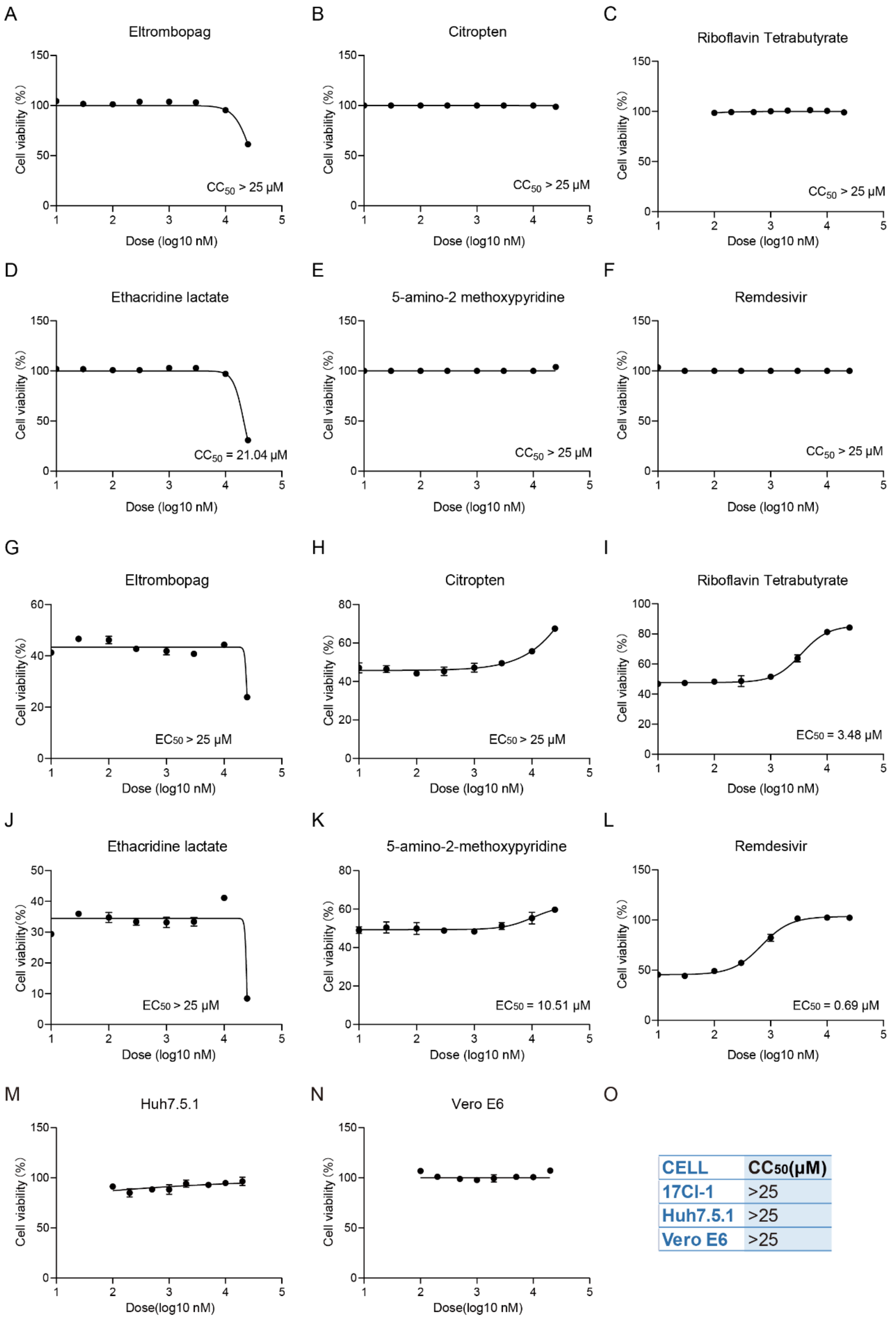

CELL	CC_{50}(μM)
17Cl-1	>25
Huh7.5.1	>25
Vero E6	>25

Figure S9. The CC_{50} and EC_{50} of 5 candidate hits.

(A-F) CCK-8 analysis of the cytotoxic effects of the top 5 hits on 17Cl-1 (24 h). (G-L) The inhibition of the top 5 hits and the value of EC_{50} is calculated according to MHV-

infected 17Cl-1 cell viability by CCK-8 (MOI = 0.1, hpi = 24 h). (M-O) CCK-8 analysis of the cytotoxic effects of RTB on Huh7.5.1 and Vero E6 (24 h).

Data were analyzed in GraphPad Prism 9.3.

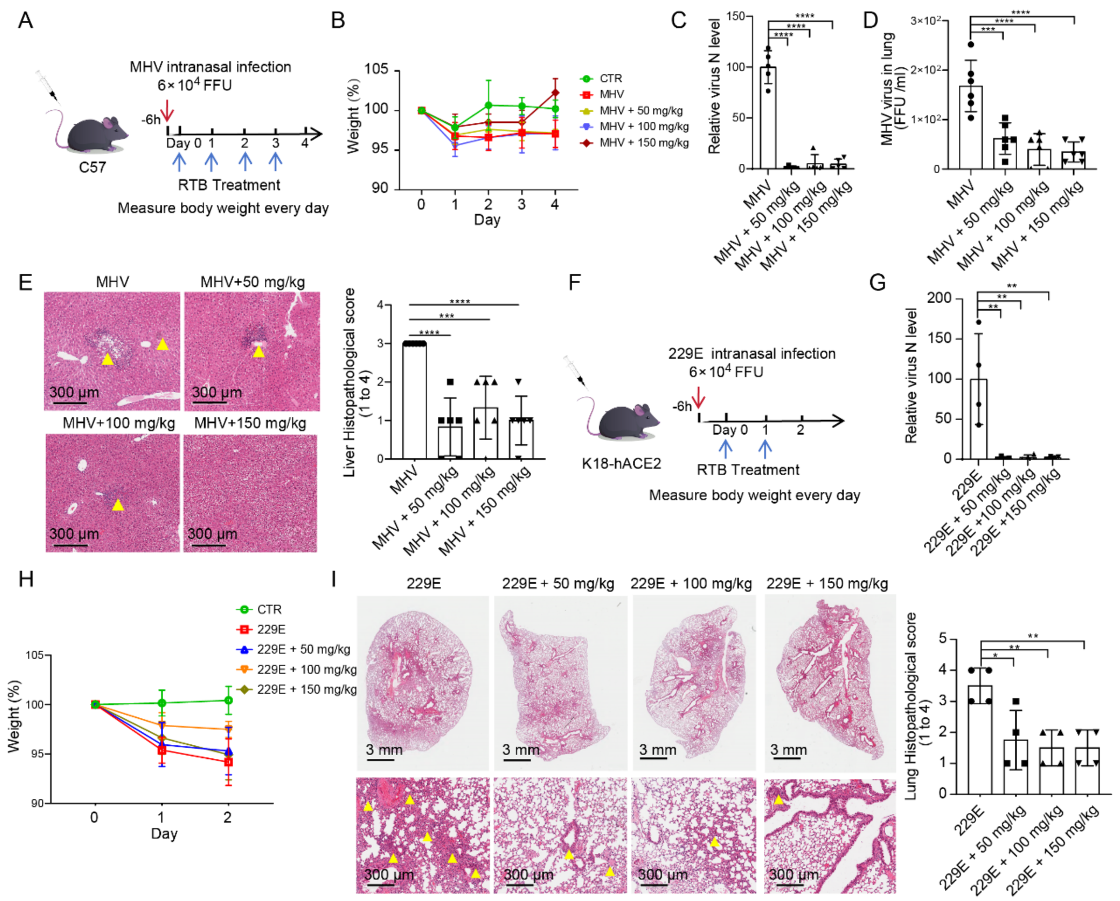

Figure S10. RTB alleviates MHV and HCoV-229E infection *in vivo*.

(A-B) Schematic illustrating MHV-infected mice treatment with RTB. MHV-infected mice were treated with PBS and RTB (50, 100, 150 mg/kg body weight) every 24 h for 4 days (A). Measure body weight for 4 days (B). (N = 6 for each group)

(C) RT-qPCR analysis of MHV viral N levels in liver of the mice treatment with PBS or RTB. *Gapdh* was used as an internal reference gene. (N = 5 for each group)

(D) Viral titration by FFA with the supernatant of homogenized lung tissues on day 4. (N = 6 for each group)

(E) HE staining analysis of liver tissue in PBS or RTB treated mice. Quantitative analysis of pathological severity scores based on the number of affected area in liver tissues. N = 9. Scale bar = 300 μm.

(F) Schematic illustrating HCoV-229E-infected K18-hACE2 mice treatment with RTB. HCoV-229E-infected mice were treated with PBS and RTB (50, 100, 150 mg/kg body weight) every 24 h for 2 days.

(G) RT-qPCR analysis of HCoV-229E viral N levels in lung of the mice treatment with PBS or RTB. *Gapdh* was used as an internal reference gene. (N = 4 for each group)

(H) Measure body weight for 2 days. (N = 4 for each group)

(I) HE staining analysis of lung tissue in PBS or RTB treated mice. Quantitative analysis

of pathological severity scores based on the number of affected area in liver tissues. (N = 4 for each group), Scale bar = 300 μm.

Data are the mean ± SD. Significance testing was performed with 1-way ANOVA and Tukey's multiple comparison test. $*P \leq 0.05$, $**P \leq 0.005$, $***P \leq 0.0005$, $****P \leq 0.0001$, ns, no significance.

A

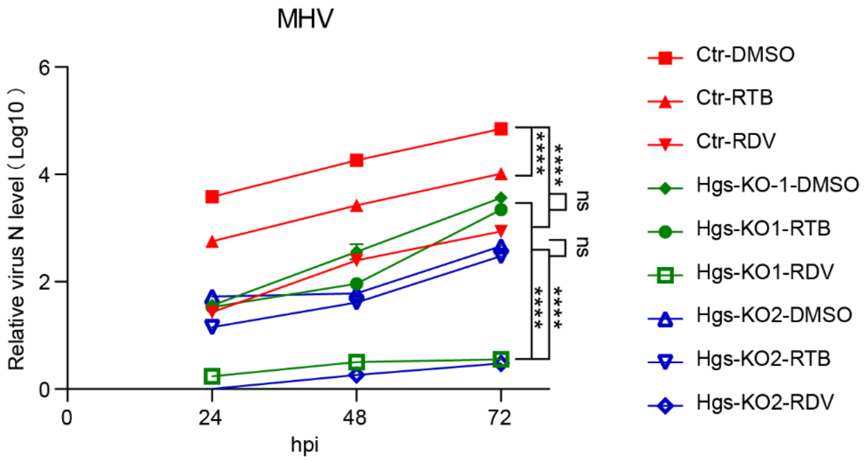

Figure S11. The anti-coronavirus activity of RTB is HGS-dependent.

RT-qPCR analysis of extracellular MHV viral gRNA levels in the Ctr, two *Hgs*-KO clones 17Cl-1 cells treated with DMSO, RTB (25 µM) or RDV (5 µM) (hpi = 24 h, 48 h, 96 h respectively, MOI = 1). N = 3 independent biological replications.

Data are the mean ± SD. Significance testing was performed with 1-way ANOVA and Tukey's multiple comparison test. *P ≤ 0.05, **P ≤ 0.005, ***P ≤ 0.0005, ****P ≤ 0.0001, ns, no significance.

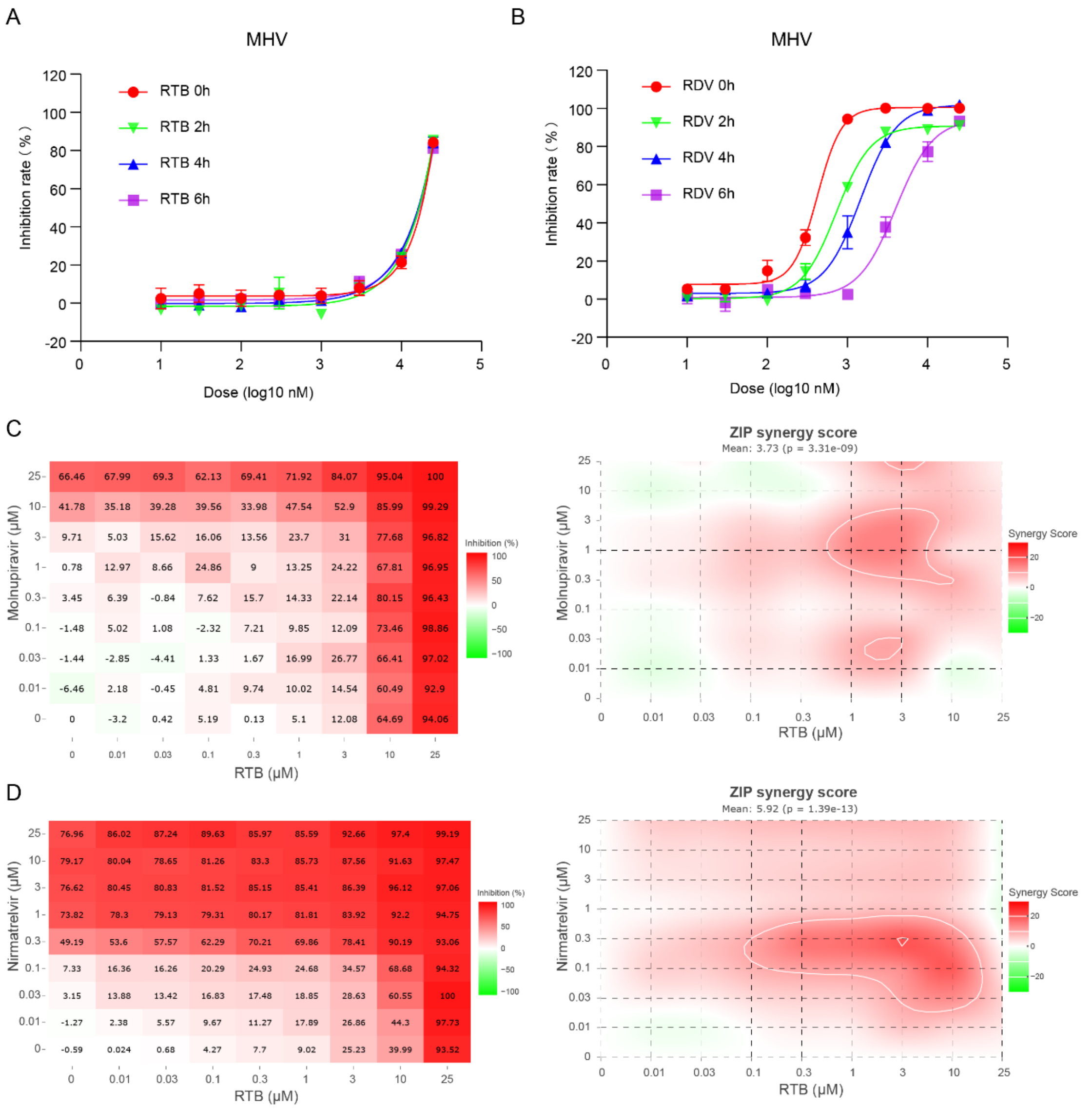

Figure S12. RTB offers an advantage over polymerase inhibitors in post-infection antiviral therapy and is ideally suited for combination therapies with current protease and polymerase inhibitors.

(A-B) Time-of-drug-addition assay. The inhibition value of RTB (25 μM) or RDV (5 μM) addition at 0, 2, 4 or 6 hpi is calculated by IFA.

(C-D) Combining RTB with Molnupiravir or Nirmatrelvir results in additive antiviral activity in vitro. An antiviral assay was performed by infecting 17Cl-1 cells with MHV and the value virus inhibition rate is calculated by IFA. Left, dose-response matrix for RTB and Molnupiravir (C) or RTB and nirmatrelvir (D) representing average %inhibition of virus replication. Right, heat map of the delta scores (%) for the same combinations and ZIP analysis where $\delta = 0$, $\delta > 0$, and $\delta < 0$ correspond to zero interaction, synergy, and antagonism, respectively. The overall zero interaction potency (ZIP) score represents the response beyond expectation (in %). In the range $-10 < ZIP < 10$, the compounds are likely to act in an additive manner, Score ≥ 10 indicate synergism. The results shown represent the means of 3 independent

experiments for each combination. Data were then analysed with the SynergyFinder webtool based on zero interaction potency (ZIP) model.

Table S2. List of Cell lines and animals.

Human: HEK293T	ATCC	Cat# CRL-3216
Human: Huh7	Cas9X^{TM}	Cat# TCH-C217
Human: Huh7.5.1	Gifted by Xiancai Ma	N/A
Mouse: 17Cl-1	Gifted by Hongyu Deng	N/A
Human: HRT-18	Gifted by Jincun Zhao	N/A
Monkey: LLC-MK2	Gifted by Jincun Zhao	N/A
African green monkey: Vero E6	Gifted by Xiancai Ma	N/A
Human: Calu-3	Gifted by Xiancai Ma	N/A
Mouse: 17Cl-1 Hgs^{KO-1}	This paper	N/A
Mouse: 17Cl-1 Hgs^{KO-2}	This paper	N/A
Mouse: 17Cl-1 Hgs^{RE-1}	This paper	N/A
Mouse: 17Cl-1 Hgs^{RE-2}	This paper	N/A
Human: Huh7.5.1 Hgs^{KO-1}	This paper	N/A
Human: Huh7.5.1 Hgs^{KO-2}	This paper	N/A
Human: Huh7.5.1 Hgs^{RE-1}	This paper	N/A
Human: Huh7.5.1 Hgs^{RE-2}	This paper	N/A
Human: HEK293T Hgs^{KO-1}	This paper	N/A
Human: HEK293T Hgs^{KO-2}	This paper	N/A
Human: HEK293T Hgs^{RE-1}	This paper	N/A
Human: HEK293T Hgs^{RE-2}	This paper	N/A
Mouse: ROSA26-Cas9	Saiye (Suzhou) Biological Information Technology Co,.Ltd	Cat# S23101012
Mouse: C57BL/6	Zhuhai BesTest Biological Technology Co,.Ltd	Cat# BST-111
Mouse: K18-hACE2-2A-CreERT2	Saiye (Suzhou) Biological Information Technology Co,.Ltd	Cat# C001244
Mouse: Hgs (flox/+) C57BL/6	Saiye (Suzhou) Biological Information Technology	Cat# S-CKO-02885

	Co,.Ltd	
HBECs	Lonza	Cat# CC-2540S

Table S3. List of PCR primers.

Gene	Sequence (5′ >>> 3′)	
Mouse *Arl8b*	Forward	GATACCCACAGTGGGCTTCAAC
	Reverse	TGCATTGACTCCTCGGCAGTAC
WIV1 *RdRp*	Forward	GGTCATGTGTGGCGGCTC
	Reverse	GCTGTAACAGCTTGACAAATGTTAAAG
nCoV *N*	Forward	CACATTGGCACCCGCAATC
	Reverse	GAGGAACGAGAAGAGGCTTG
OC43 *N*	Forward	GGGACCCAAGTAGCGATGAG
	Reverse	TGGCTCTACTACGCGATCCT
229E *N*	Forward	CGCAAGAATTCAGAACCAGAG
	Reverse	GGGAGTCAGGTTCTTCAACAA
NL63 *N*	Forward	AGGACCTTAAATTCAGACAACGTTCT
	Reverse	GATTACGTTTGCGATTACCAAGACT
MHV *N*	Forward	TGGAAGGTCTGCACCTGCTA
	Reverse	TTTGGCCCACGGGATTG
MHV *ORF1a*	Forward	GTTCAGTCTGCCATAATCCG
	Reverse	GTCACAAGAGCACTCCCTG
MHV *ORF1b*	Forward	GAGTATGCCTCCAACTCTGC
	Reverse	ACAAACGGAAGCACCACC
HSV-1 *ICP0*	Forward	CCCACTATCAGGTACACCAGCTT
	Reverse	CTGCGCTGCGACACCTT
Human *GAPDH*	Forward	GTCTCCTCTGACTTCAACAGCG
	Reverse	ACCACCCTGTTGCTGTAGCCAA
Mouse *GAPDH*	Forward	CATCACTGCCACCCAGAAGACTG
	Reverse	ATGCCAGTGAGCTTCCCGTTCAG
Cre recombinase	Forward	CATATTGGCAGAACGAAAACGC
	Reverse	CCTGTTTCACTATCCAGGTTACGG
HGS loxP1	Forward	AACCCTTAGAGGGAAACGCAAATC
	Reverse	TCTAGGGTCCACAGCTAAACTCTC
HGS loxP2	Forward	CTGCTGCAAGGCTACAAGAGT
	Reverse	ATCCTAATCCATGATCCCCTTTCT

Table S4. List of Chemicals, compounds, peptides, and recombinant proteins.

Hoechst 33342	Thermo Fisher	Cat# H1399
Puromycin Dihydrochloride	Beyotime Biotechnology	Cat# ST551
Blasticidin S HCl	Beyotime Biotechnology	Cat# ST018
Hygromycin B	Beyotime Biotechnology	Cat# ST1389
Polybrene (Hexadimethrine Bromide)	Beyotime Biotechnology	Cat# C0351
Tamoxifen	Sigma-Aldrich	Cat# 10540-29-1
Protein A/G Agarose Beads	Proteintech	Cat# PR40025
Anti FLAG Nanobody Magarose Beads	KTSM-Life	Cat# KTSM1338
Glutathione Sepharose (GST Tag)	Cytiva	Cat# 17075605
Lipofectamine™ 3000 Transfection Reagent	Thermo Fisher	Cat# L3000015
TRIzol™ Reagent	Invitrogen	Cat# 15596018
Fetal Bovine Serum, Premium Plus	Thermo Fisher	Cat# A5669701
TrypLE™ Express Enzyme (1 ×), phenol red	Thermo Fisher	Cat# 12605028
ACCUTASE™	STEMCELL Technologies	Cat# 07920
Opti-MEM® I Reduced Serum Medium	Gibco	Cat# 31985070
Penicillin-Streptomycin, Liquid (100 ×)	Invitrogen	Cat# 15140122
EMS Aqueous Glutaraldehyde EM Grade 25%	Electron Microscopy Sciences	Cat# 16220
Peptide FLAG: GRKKRRQRRRPPQDYKDDDDK	DGpeptides Co., Ltd	N/A
Membrane (M) peptide M146 : GRKKRRQRRRPPQESELVIGAVILR	DGpeptides Co., Ltd	N/A
Membrane (M) peptide M146-DRI: RLIVAGIVLESEQPPRRRQRRKKRG	DGpeptides Co., Ltd	N/A
FITC-Membrane (M) peptide M146-DRI: FITC-RLIVAGIVLESEQPPRRRQRRKKRG	DGpeptides Co., Ltd	N/A

Membrane (M) peptide M161: GRKKRRQRRRPPQESELVIGAVILRGHLRIAG	DGpeptides Co., Ltd	N/A
Membrane (M) peptide M161-DRI: GAIRLHGRLIVAGIVLESEQPPRRRQRRKKRG	DGpeptides Co., Ltd	N/A
Membrane (M) M146-E4： GRKKRRQRRRPPQESELVIGAVILREEEE	DGpeptides Co., Ltd	N/A
Membrane (M) M146-K4： GRKKRRQRRRPPQESELVIGAVILRKKKK	DGpeptides Co., Ltd	N/A
Membrane (M) M161-P1： GRKKRRQRRRPPQESELPIGAVILRGHLRIAG	DGpeptides Co., Ltd	N/A
Membrane (M) M161-P2： GRKKRRQRRRPPQESELPPGAVILRGHLRIAG	DGpeptides Co., Ltd	N/A
DYKDDDDK tag Fusion protein (FLAG-GST)	Proteintech	Cat# Ag2329
2019-nCOV Spike protein (RBD) Fusion Protein (GST-RBD)	Proteintech	Cat# Ag30689
Membrane glycoprotein Fusion Protein (GST-M :101-222 aa)	Proteintech	Cat# Ag30691
Immobilon NC Transfer Membrane	Merck millipore	Cat# HATF00010
Peptide FLAG-M	This paper	N/A
Peptide HIS-HGS	This paper	N/A
Peptide HIS-HGS(1-390)	This paper	N/A
Riboflavin Tetrabutyrate	Selleck	S6441
Nirmatrelvir (PF-07321332)	Selleck	S9866
Molnupiravir (EIDD-2801)	Selleck	S8969
Remdesivir (GS-5734)	TargetMol	T7766

Table S5. List of sgRNA primer sequence.

**Name**	**Sequence (5′ to 3′)**
Mouse *Inppl1* target (KD)	ACCCGAGTCGCCCTAGAGG
Mouse *Pdzd8* target (KD)	CCGCTCGGGCCGCCCCCGG
Mouse *Hmgcr* target (KD)	GCGTCCGCCAGCTCACCTC
Mouse *Hgs* target (KD)	CTGCAGCGTCGGTCCGGAG
Mouse *Agtr2* target (KD)	CTCAGAGGCTGGCGATGGA
Mouse *Copa* target (KD)	GAAGCCTTGGGAGTGCCAC
Mouse *Shmt2* target (KD)	CTAGCCACTAACTCTGTAT
Mouse *Mtmr4* target (KD)	CAGCTGCGGGAGACGGAAG
Mouse *Hcrt* target (KD)	AGCACTGAGAGAGGAGTAT
Mouse *F13a1* target (KD)	GGCAAAGTGACCAGAAAGT
Mouse *Camk1* target (KD)	CTGGCTGCGAGGGCCGAGG
Mouse *Dusp6* target (KD)	ACTGGGTAGGAACAAAACT
Mouse *Prkrir* target (KD)	TCCCCTCCCCGGCCCCGCG
Mouse *Csnk1g3* target (KD)	CCGGAAGTGAACACATGAG
Mouse *Ramp3* target (KD)	CAGCAACAGCGACATTCGG
Mouse *Olfr873* target (KD)	GAAATAACCCGAAGAGAAT
Mouse *Iars2* target (KD)	GCAGCCGCCCGGTACCTAA
Mouse *Olfr524* target (KD)	GAACAGCCCTAGAAGGCTT
Mouse *Sod1* target (KD)	GCCTCCCCGCGCCCCGGAG
Mouse *Got1* target (KD)	TGTACACCGGAGGAGGTGT
Mouse *Syngr2* target (KD)	CGAGGCGCGTCACCACCTG
Mouse *Extl2* target (KD)	GAGGGAGGGGTGGCCACTC
Mouse *Atp13a2* target (KD)	TCCGCGGAGGGGCCGGGCT
Mouse *Zyg11b* target (KD)	GCTCCAGGAGGCTCGGGTC
Mouse *Arl8b* target (KD)	GGGCCGATACCCGCTCGT
Mouse *Hgs* target # 1 (KO)	TCTGCGACCTGATCCGTCAG
Mouse *Hgs* target # 2 (KO)	TCCGGAACGAACCCAAGTAC
Human *Hgs* target # 1 (KO)	GTACACTTCGTACCCCAAGG
Human *Hgs* target # 2 (KO)	ACTCTTCATGCGGTTCACGA
Monkey *Hgs* target # 1 (KO)	CTTGGGGTACGAAGTGTACG
Monkey *Hgs* target # 2 (KO)	CCGGAATGAGCCTAAGTACA

Table S6. List of antibodies.

Antibodies		
APC anti-human CD107a (LAMP-1) Antibody	BioLegend	Cat# 328620<br><br>(FC:1/100)
FITC anti-human CD107a (LAMP-1) Antibody	BioLegend	Cat# 328606<br><br>(FC:1/100)
CoraLite® Plus 488 Anti-Mouse CD107a / LAMP1 (1D4B)	Proteintech	(IF:1/100)
β-actin Mouse Monoclonal Antibody	Beijing Ray Antibody Biotech	Cat# RM2001-IR<br><br>(WB:1/5000)
HRS (D7T5N) Rabbit mAb (Hgs)	Cell Signaling Technology	Cat# 15087S<br><br>(WB:1/2000; IF:1/500; IP:1/250)
CoraLite® Plus 488-conjugated HGS Polyclonal antibody	Proteintech	Cat# CL488-10390<br><br>(IF:1/100)
ERGIC53 Rabbit pAb A10440	Abclonal	Cat# A10440<br><br>(IF:1/100)
GOLGA2/GM130 Monoclonal antibody	Proteintech	Cat# 66662-1-Ig-100ul<br><br>(IF:1/100)
β-tubulin Mouse Monoclonal Antibody	HUABIO	Cat# EM0103<br><br>(WB:1/5000)
GST Tag Monoclonal antibody	Proteintech	Cat# 66001-2-1g<br><br>(WB:1/2000)
Anti-HA-tag mAb	MBL	Cat# M180-3<br><br>(WB:1/2000; IF:1/250)
DYKDDDDK Tag Monoclonal Antibody	Proteintech	Cat# 66008-4-1g<br><br>(WB:1/2000; IF:1/250)
GFP-tag Mouse Monoclonal Antibody	Ray Antibody	Cat# RM1008

		(WB:1/5000)
SARS-CoV / SARS-CoV-2 (COVID-19) spike antibody [1A9]	Genetex	Cat# GTX632604-S<br><br>(WB:1/2000)
SARS-CoV-2 S protein (944-1214 aa) Polyclonal antibody	Proteintech	Cat# 28867-1-AP<br><br>(IF:1/100)
SARS-CoV-2 (COVID-19) Membrane antibody [HL1088]	Genetex	Cat# GTX636246-S<br><br>(WB:1/2000; IF:1/250)
SARS-CoV/SARS-CoV-2 Nucleocapsid Antibody, Rabbit MAb	Sino Biological	Cat# 40143-R004<br><br>(WB:1/2000)
SARS-CoV-2 (COVID-19) Nucleocapsid antibody, Human MAb	This paper	(IF:1/100)
Human coronavirus (HCoV-HKU1) Nucleocapsid Antibody, Rabbit PAb	Sino Biological	Cat# 40642-T62<br><br>(WB:1/1000; IF:1/100)
Human coronavirus (HCoV-OC43) Nucleocapsid Antibody, Rabbit PAb	Sino Biological	Cat# 40643-T62<br><br>(WB:1/1000; IF:1/100)
Human coronavirus (HCoV-229E) Nucleocapsid Antibody, Rabbit PAb	Sino Biological	Cat# 40640-T62<br><br>(WB:1/1000; IF:1/100)
Human coronavirus (HCoV-NL63) Nucleocapsid Antibody, Rabbit PAb	Sino Biological	Cat# 40641-T62<br><br>(WB:1/1000; IF:1/100)
MERS-CoV Nucleocapsid Protein Antibody, Rabbit PAb	Sino Biological	Cat# 40068-RP02<br><br>(WB:1/1000)
SARS-CoV-2 (COVID-19) Envelope antibody [HL1443]	Genetex	Cat# GTX636915-

		S<br><br>(WB:1/1000)
MHV Nucleocapsid Antibody, Mouse MAb [2E6]	Gifted by Rong Ye	N/A
Alexa Fluor 488 Anti-Cytokeratin 5 antibody [EP1601Y]	Abcam	Cat# ab193894<br><br>(IF:1/400)
Anti-alpha Tubulin (acetyl K40) antibody [6-11B-1]	Abcam	Cat# ab24610
MUC5AC Monoclonal Antibody (45M1)	Thermo Fisher	Cat# AC011
Mouse Control IgG	Abclonal	Cat# AC011
Rabbit Control IgG	Abclonal	Cat# AC005
Goat anti-Mouse IgG (H+L) Highly Cross-Adsorbed Secondary Antibody, Alexa Fluor Plus 488	Invitrogen	Cat# A32723
Goat anti-Rabbit IgG (H+L) Highly Cross-Adsorbed Secondary Antibody, Alexa Fluor Plus 488	Invitrogen	Cat# A32731
Goat anti-Mouse IgG (H+L) Cross-Adsorbed Secondary Antibody, Alexa Fluor 568	Invitrogen	Cat# A11004
Goat anti-Rabbit IgG (H+L) Cross-Adsorbed Secondary Antibody, Alexa Fluor 568	Invitrogen	Cat# A11011
Goat anti-Mouse IgG (H+L) Highly Cross-Adsorbed Secondary Antibody, Alexa Fluor Plus 647	Invitrogen	Cat# A32728
Goat anti-Rabbit IgG (H+L) Highly Cross-Adsorbed Secondary Antibody, Alexa Fluor Plus 647	Invitrogen	Cat# A32733
Goat anti-Human IgG (H+L) Cross-Adsorbed Secondary Antibody, Alexa Fluor 488	Invitrogen	Cat# A11013
IRDye® 800CW Goat anti-Rabbit IgG Secondary Antibody	LI-COR	Cat# 926-32211
IRDye® 680RD Goat anti-Mouse IgG (H+L)	LI-COR	Cat# 926-68070

Table S7. List of Critical commercial assay.

EasyPure® Simple Viral DNA/RNA Kit	TransGen Biotech	Cat# ER211-01
NucleoBond Xtra Midi (50)	MACHEREY-NAGEL	Cat# 740410.50
ClonExpress® Ultra One Step Cloning Kit	Vazyme Biotech	Cat# C115-01
Phanta® Max Super-Fidelity DNA Polymerase	Vazyme Biotech	Cat# P505-d2
SuperScript™ IV First-Strand Synthesis System	Invitrogen&trade	Cat# 18091050
Cell Counting Kit-8	Beyotime	Cat# C0039
BD Cytofix/Cytoperm™ solution	BD Bioscience	Cat# 554722
1 × BD Perm/Wash buffer	BD Bioscience	Cat# 554723
NucleoSpin® Blood	MACHEREY-NAGEL	Cat# 740951.50
