# Supplementary figures and images for: Targeting the host factor HGS–viral membrane protein interaction in coronavirus infection
Source: J Clin Invest. 2025 Dec 16;136(5):e200225. doi: 10.1172/JCI200225 (PMC12948427; doi:10.1172/JCI200225)

Figure 3

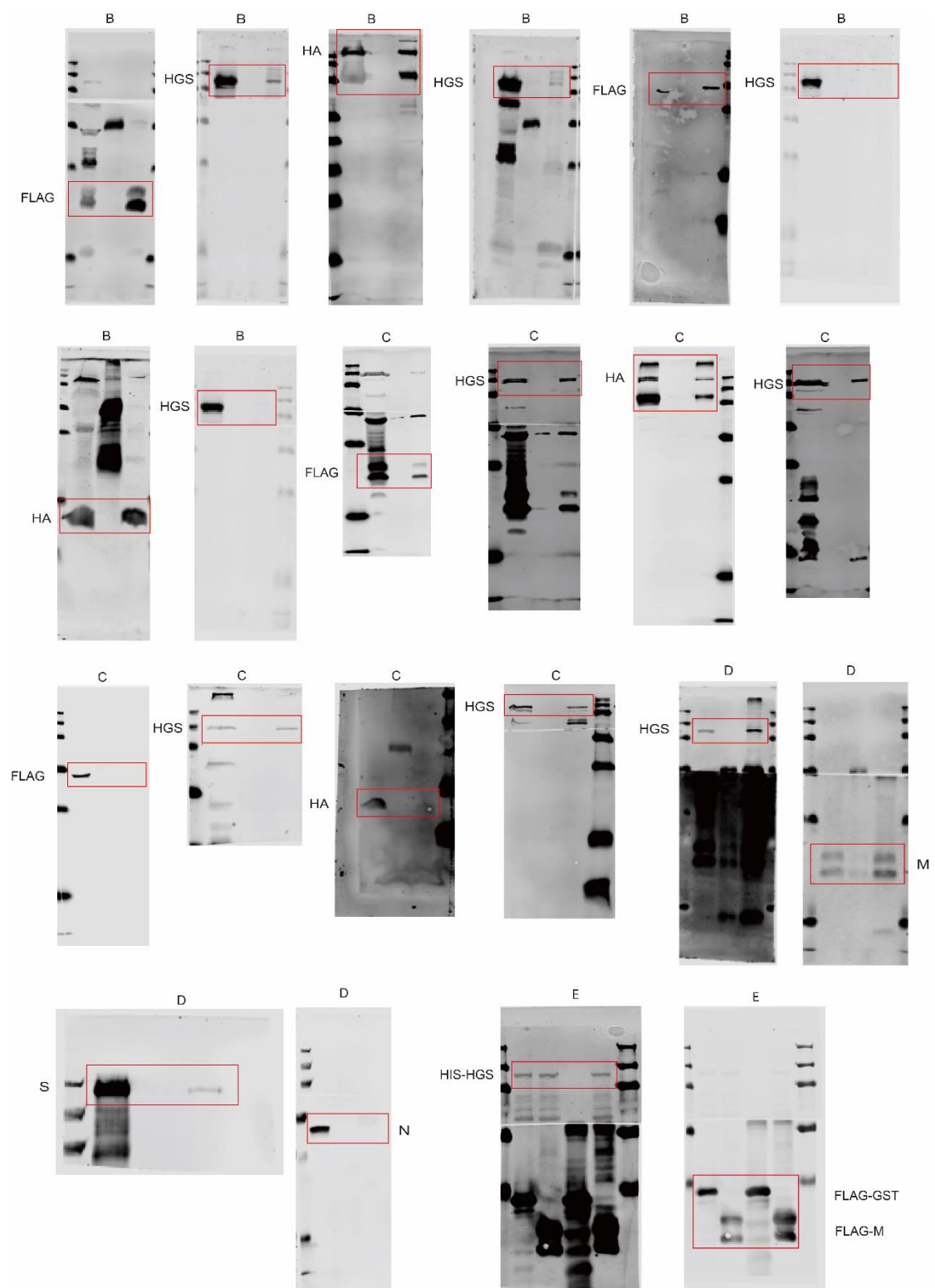

Figure 7

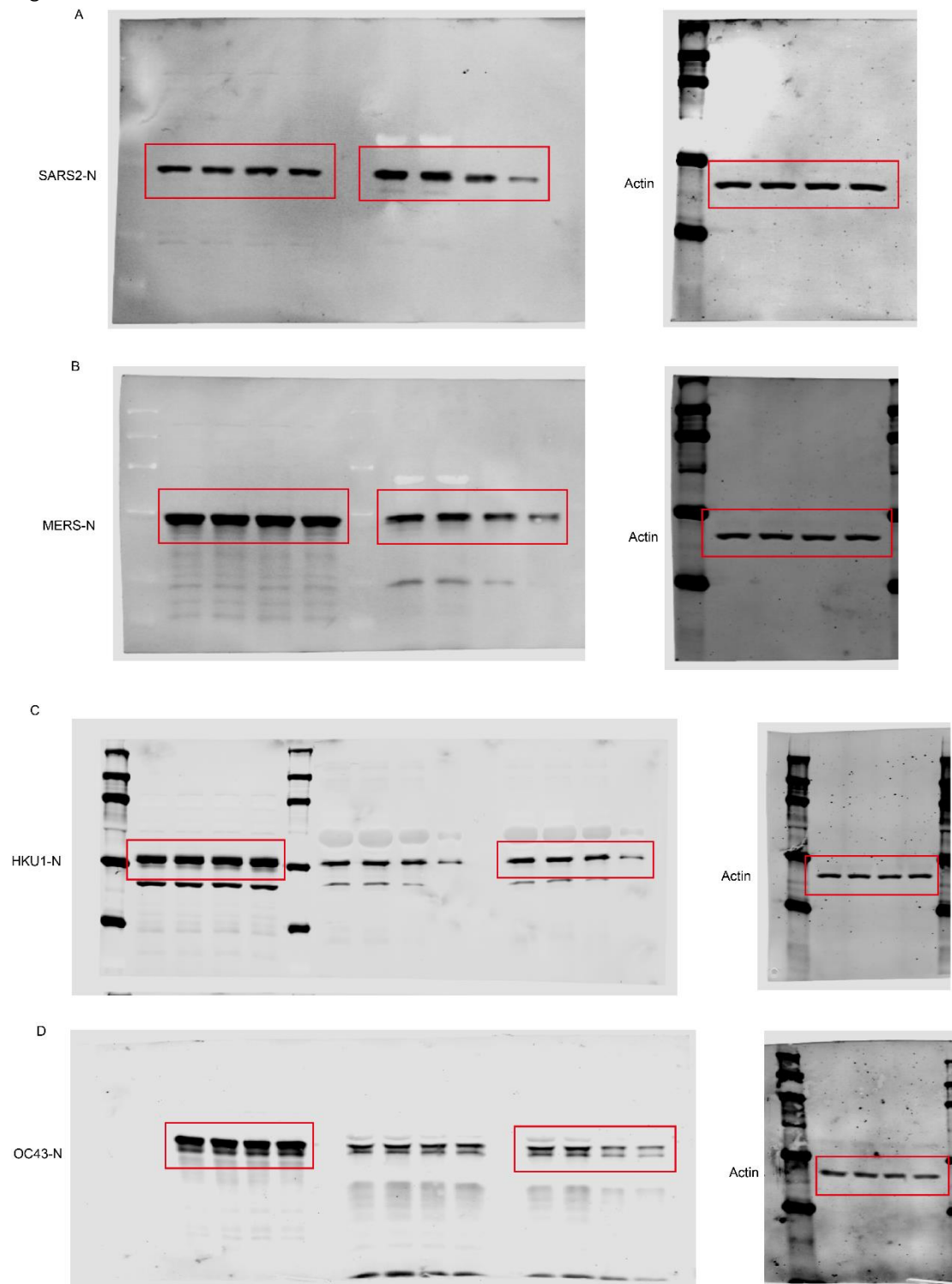

Figure S2

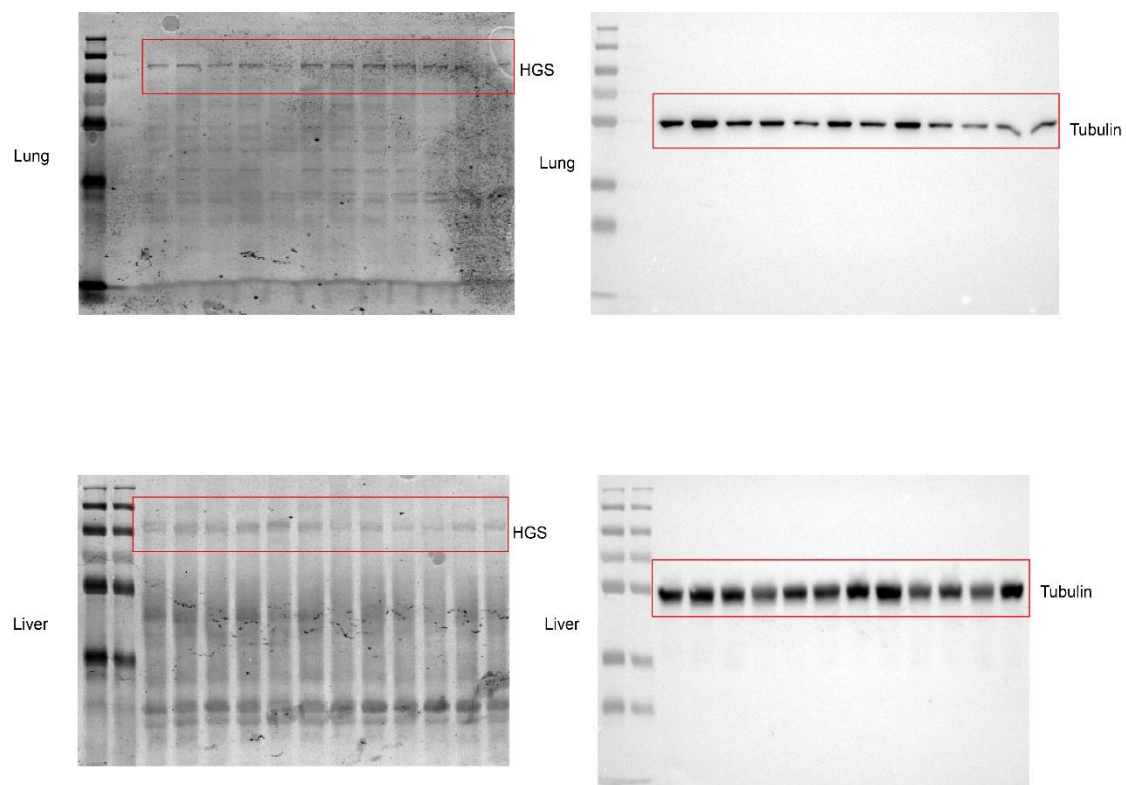

Figure S3

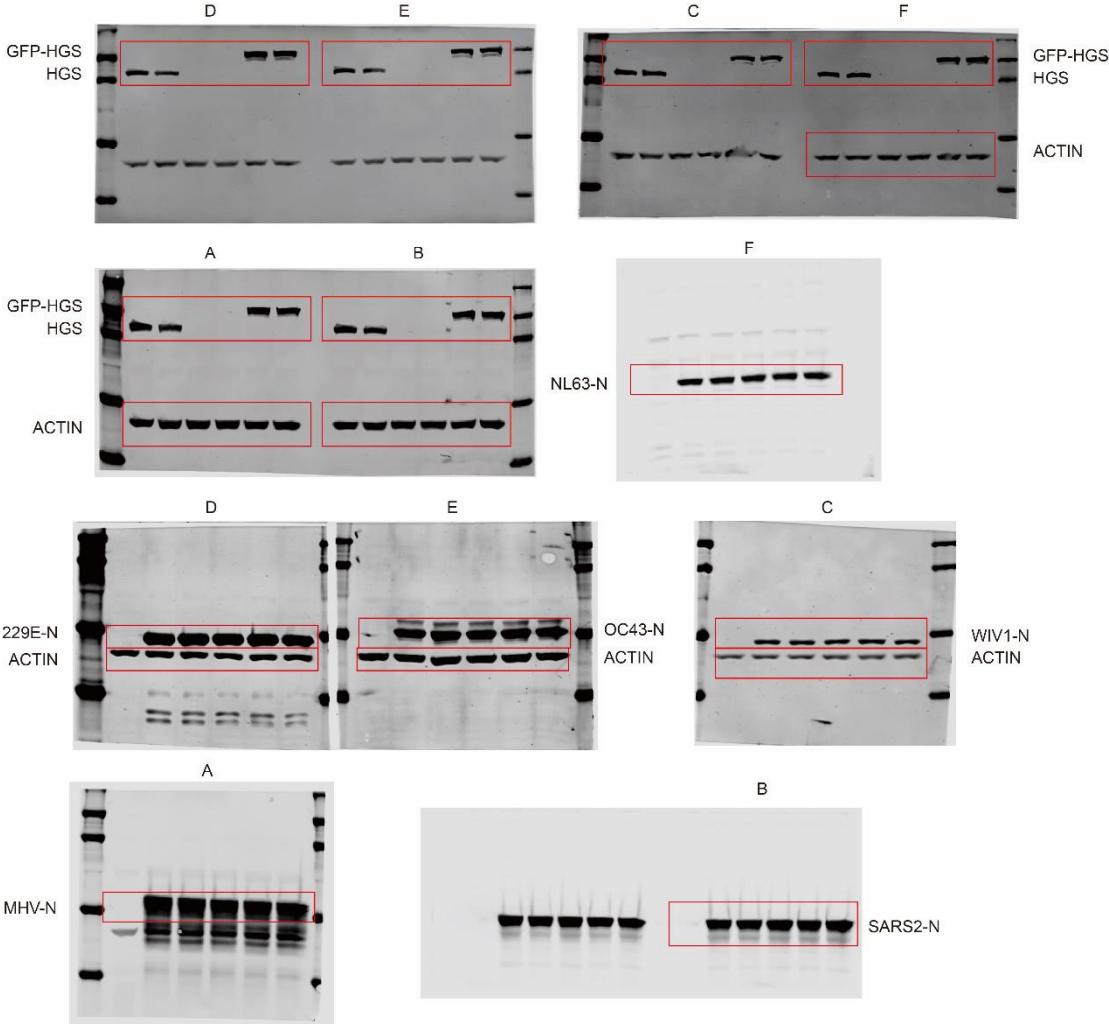

Figure S6

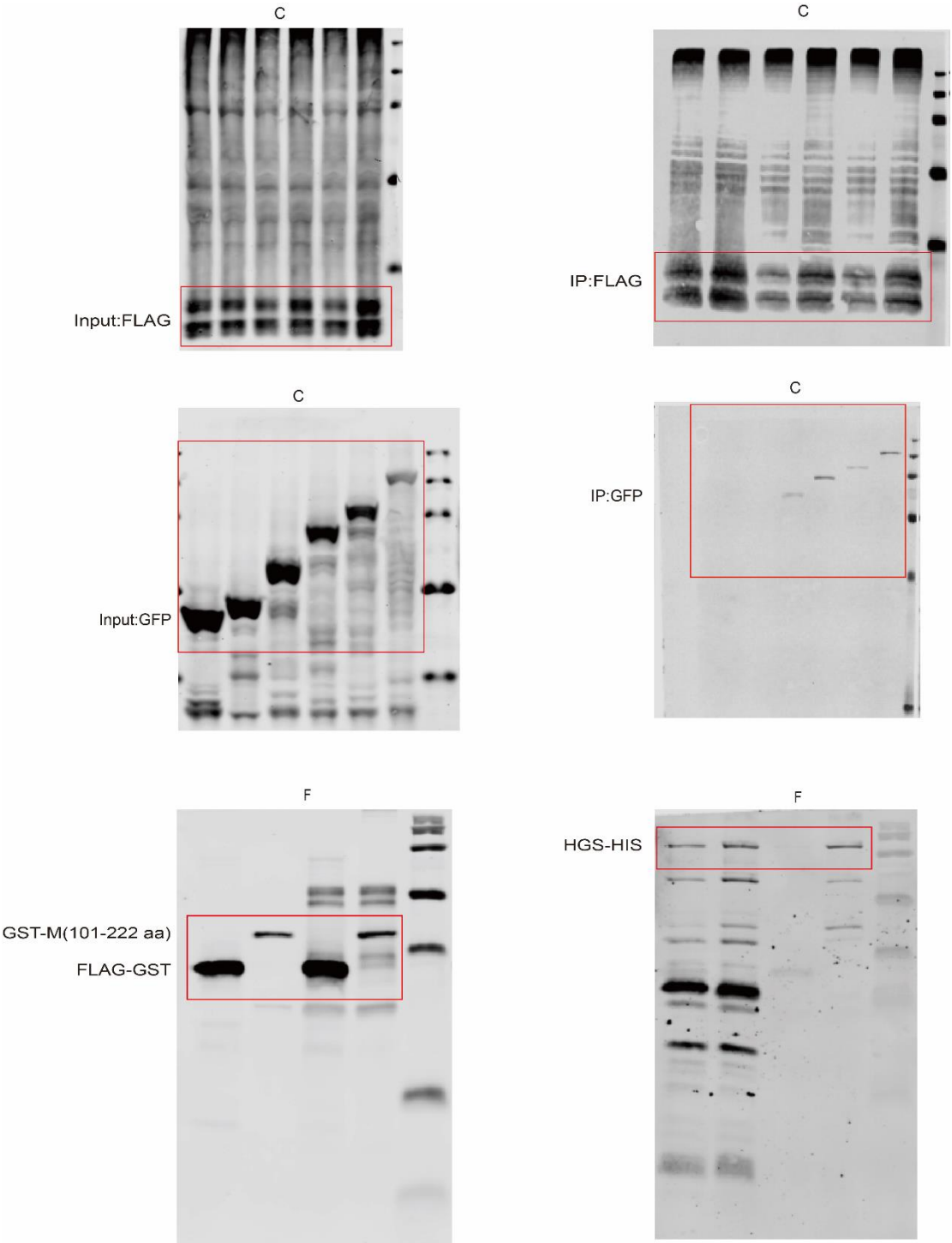

Figure S7

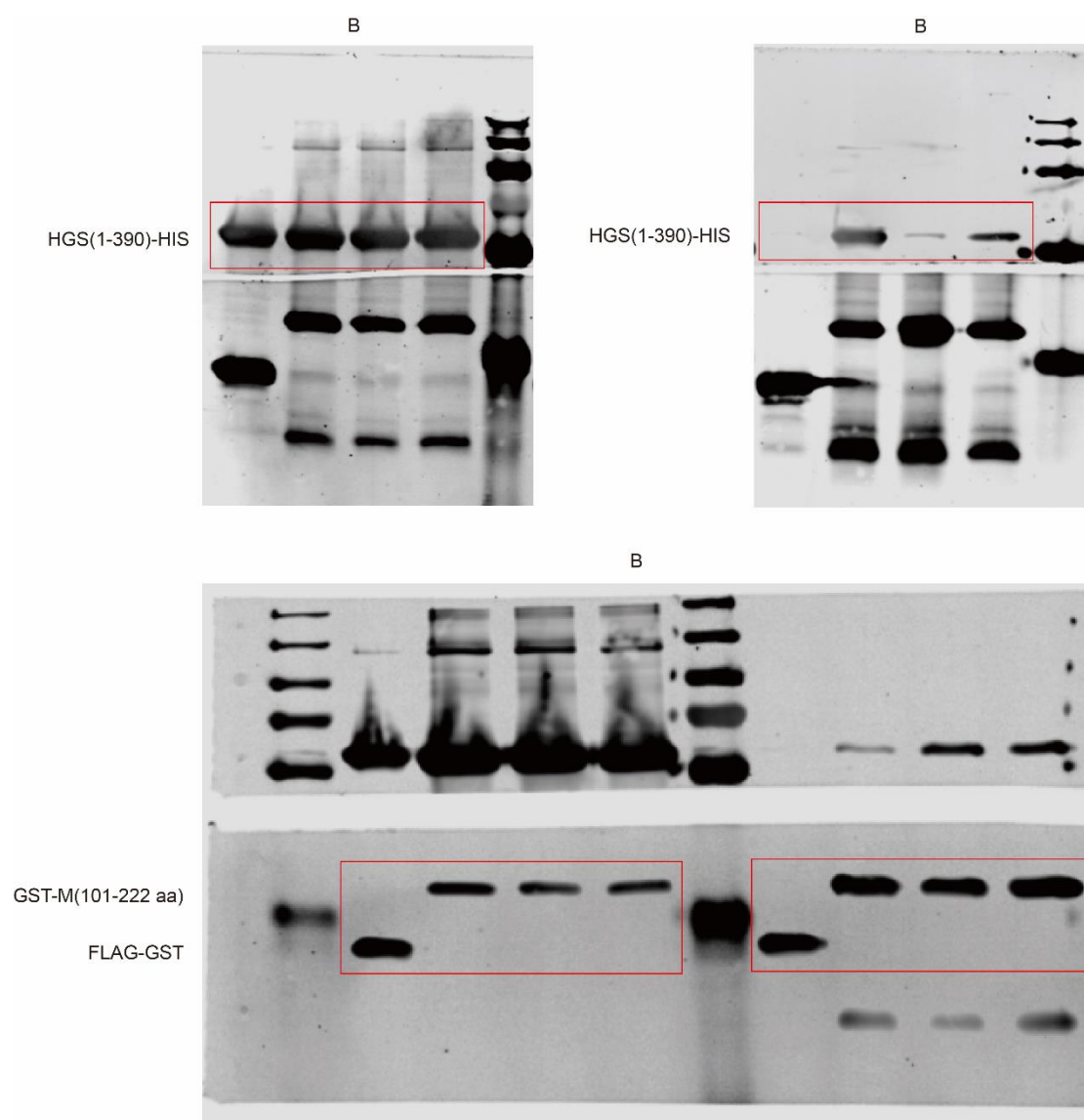

Supplement: Unedited blot and gel images [file jci-136-200225-s270.pdf]
